# Supplementary material for: Diversity and Advantages of Culturable Endophytic Fungi from Tea (Camellia sinensis)
Source: J Fungi (Basel). 2023 Dec 13;9(12):1191. doi: 10.3390/jof9121191 (PMC10744531; doi:10.3390/jof9121191)
Supplement: Supplementary file 1 [file jof-09-01191-s001.zip › jof-2691500-supplementary.pdf]

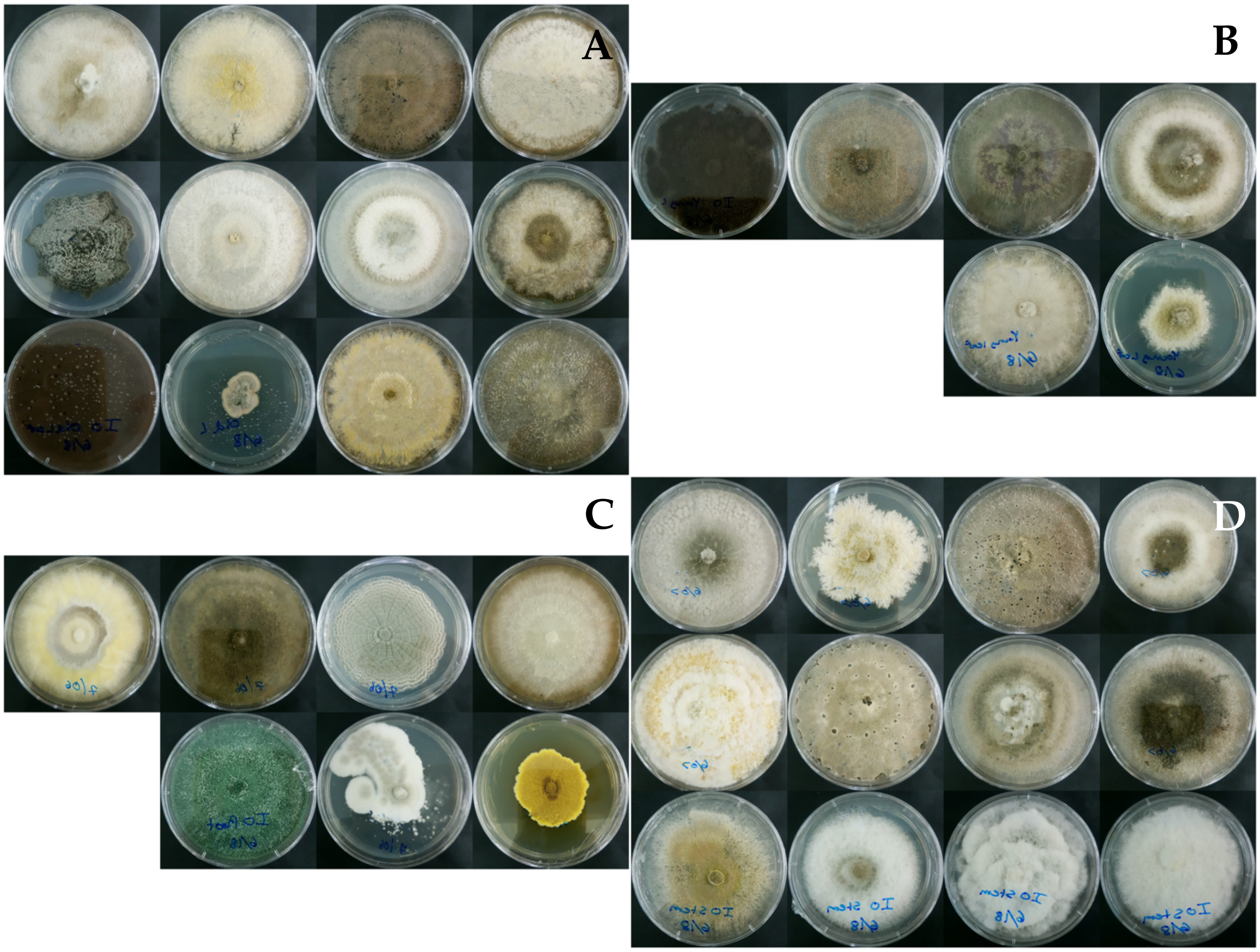

Supplementary Figure S1. Representative of fungal strains that were isolated from tea grown in the conventional system including six strains from young leaves (A), 12 strains from old leaves (B), 12 strains from stem (C), and seven strains from root (D). All fungal strains were cultivated on PDA media for 7 days at 28 ^{o}C.

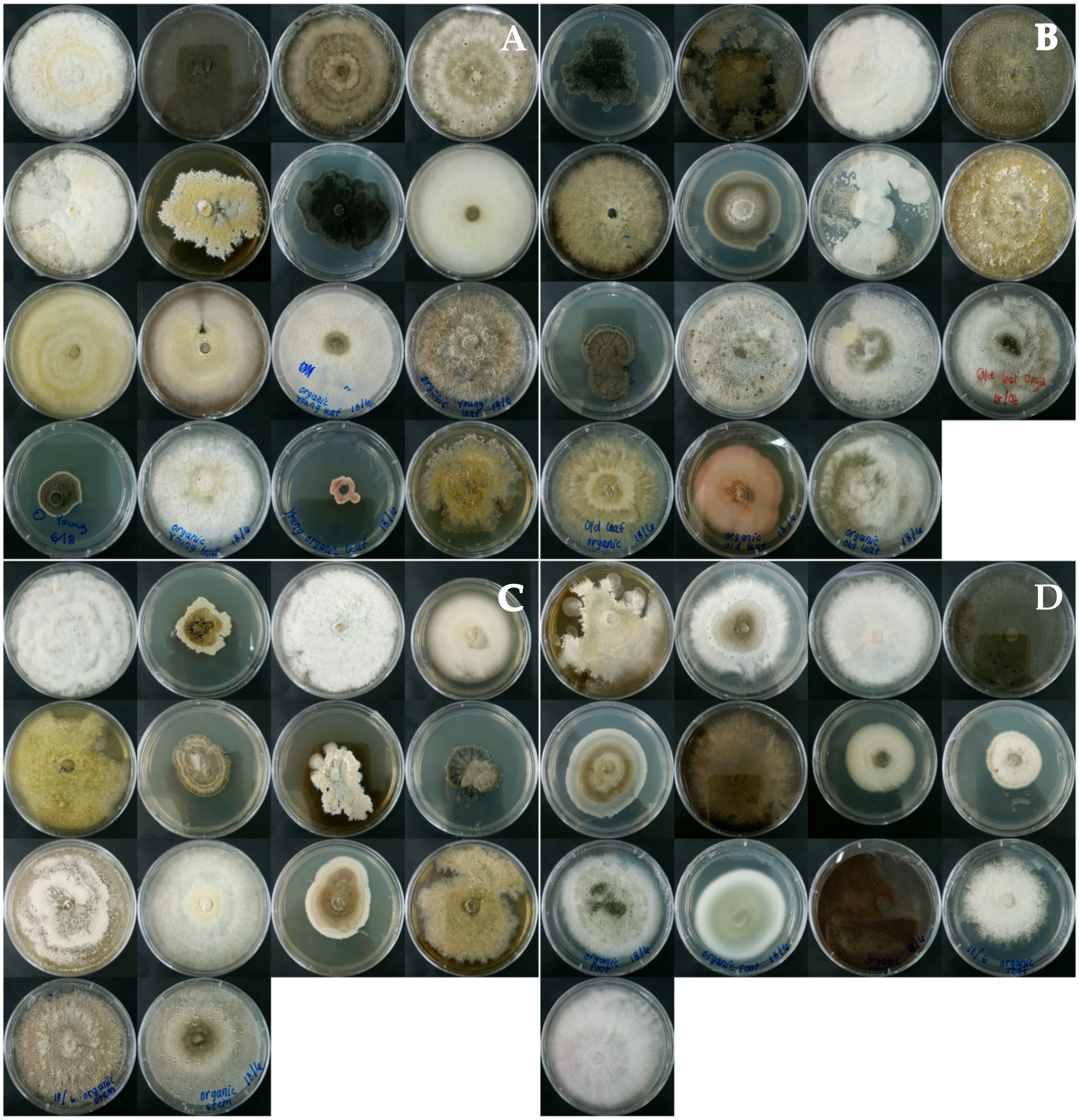

Supplementary Figure S2. Representative of fungal strains that were isolated from tea grown in the organic system including 16 strains from young leaves (A), 15 strains from old leaves (B), 14 strains from stem (C), and 13 strains from root (D). All fungal strains were cultivated on PDA media for 7 days at 28 ^{o}C.

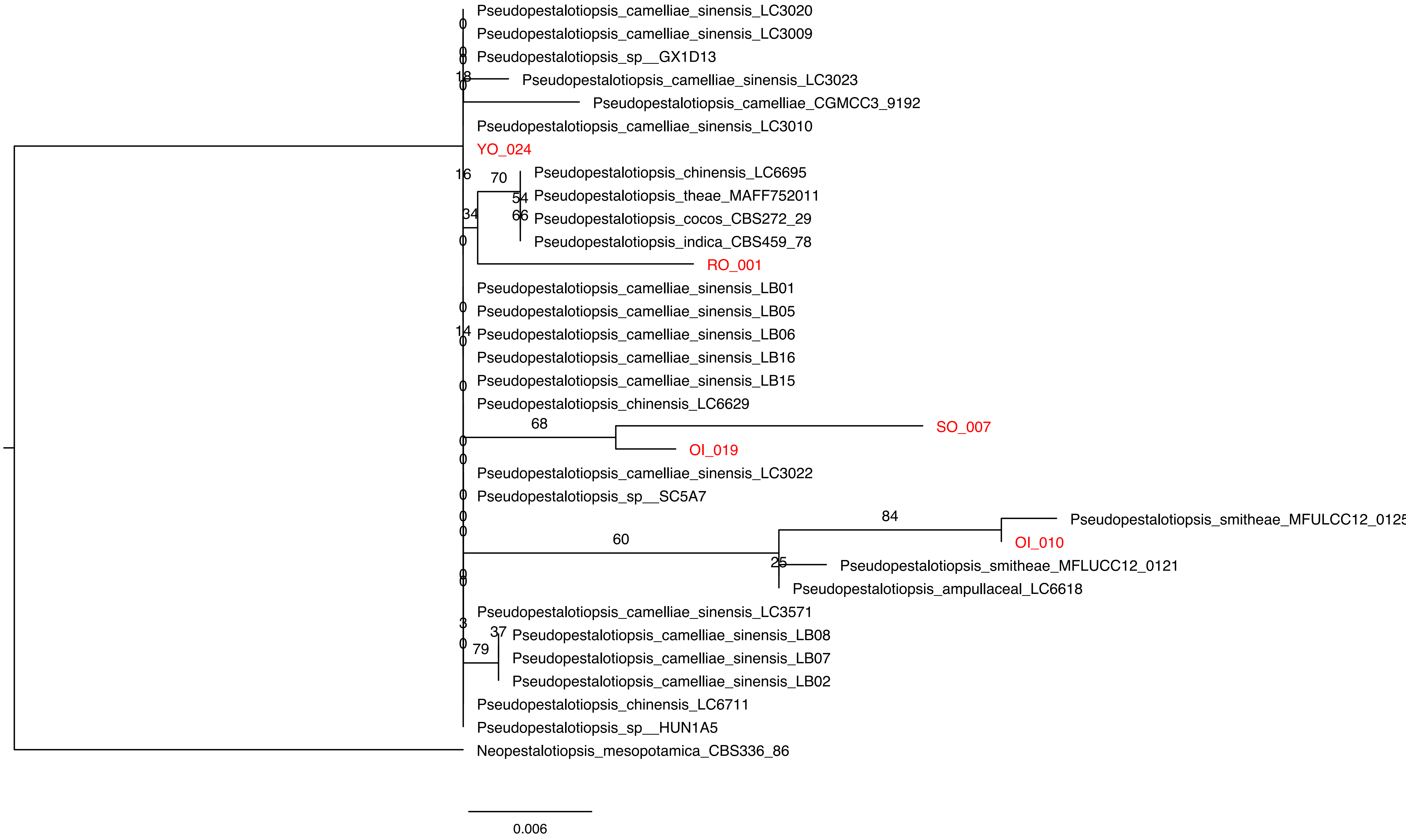
Pseudopestalotiopsis_camelliae_sinensis_LC3020
Pseudopestalotiopsis_camelliae_sinensis_LC3009
Pseudopestalotiopsis_sp__GX1D13
Pseudopestalotiopsis_camelliae_sinensis_LC3023
Pseudopestalotiopsis_camelliae_CGMCC3_9192
Pseudopestalotiopsis_camelliae_sinensis_LC3010
YO_024
Pseudopestalotiopsis_chinensis_LC6695
Pseudopestalotiopsis_theae_MAFF752011
Pseudopestalotiopsis_cocos_CBS272_29
Pseudopestalotiopsis_indica_CBS459_78
RO_001
Pseudopestalotiopsis_camelliae_sinensis_LB01
Pseudopestalotiopsis_camelliae_sinensis_LB05
Pseudopestalotiopsis_camelliae_sinensis_LB06
Pseudopestalotiopsis_camelliae_sinensis_LB16
Pseudopestalotiopsis_camelliae_sinensis_LB15
Pseudopestalotiopsis_chinensis_LC6629
SO_007
OI_019
Pseudopestalotiopsis_camelliae_sinensis_LC3022
Pseudopestalotiopsis_sp__SC5A7
Pseudopestalotiopsis_smitheae_MFULCC12_0125
OI_010
Pseudopestalotiopsis_smitheae_MFLUCC12_0121
Pseudopestalotiopsis_ampullaceal_LC6618
Pseudopestalotiopsis_camelliae_sinensis_LC3571
Pseudopestalotiopsis_camelliae_sinensis_LB08
Pseudopestalotiopsis_camelliae_sinensis_LB07
Pseudopestalotiopsis_camelliae_sinensis_LB02
Pseudopestalotiopsis_chinensis_LC6711
Pseudopestalotiopsis_sp__HUN1A5
Neopestalotiopsis_mesopotamica_CBS336_86
0.006

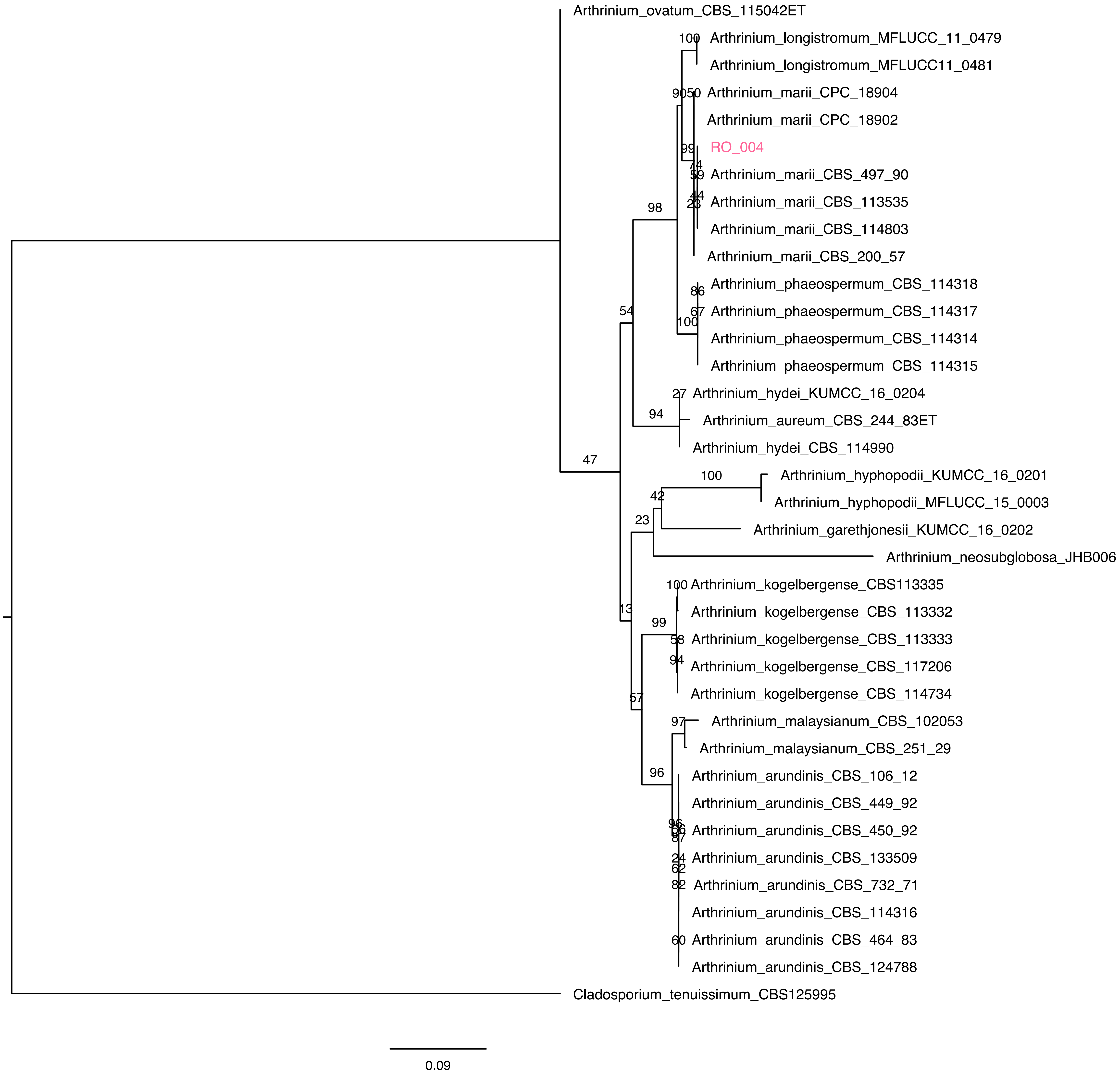
Arthrinium_ovatum_CBS_115042ET
Arthrinium_longistromum_MFLUCC_11_0479
Arthrinium_longistromum_MFLUCC11_0481
Arthrinium_marii_CPC_18904
Arthrinium_marii_CPC_18902
RO_004
Arthrinium_marii_CBS_497_90
Arthrinium_marii_CBS_113535
Arthrinium_marii_CBS_114803
Arthrinium_marii_CBS_200_57
Arthrinium_phaeospermum_CBS_114318
Arthrinium_phaeospermum_CBS_114317
Arthrinium_phaeospermum_CBS_114314
Arthrinium_phaeospermum_CBS_114315
Arthrinium_hydei_KUMCC_16_0204
Arthrinium_aureum_CBS_244_83ET
Arthrinium_hydei_CBS_114990
Arthrinium_hyphopodii_KUMCC_16_0201
Arthrinium_hyphopodii_MFLUCC_15_0003
Arthrinium_garethjonesii_KUMCC_16_0202
Arthrinium_neosubglobosa_JHB006
Arthrinium_kogelbergense_CBS113335
Arthrinium_kogelbergense_CBS_113332
Arthrinium_kogelbergense_CBS_113333
Arthrinium_kogelbergense_CBS_117206
Arthrinium_kogelbergense_CBS_114734
Arthrinium_malaysianum_CBS_102053
Arthrinium_malaysianum_CBS_251_29
Arthrinium_arundinis_CBS_106_12
Arthrinium_arundinis_CBS_449_92
Arthrinium_arundinis_CBS_450_92
Arthrinium_arundinis_CBS_133509
Arthrinium_arundinis_CBS_732_71
Arthrinium_arundinis_CBS_114316
Arthrinium_arundinis_CBS_464_83
Arthrinium_arundinis_CBS_124788
Cladosporium_tenuissimum_CBS125995
0.09

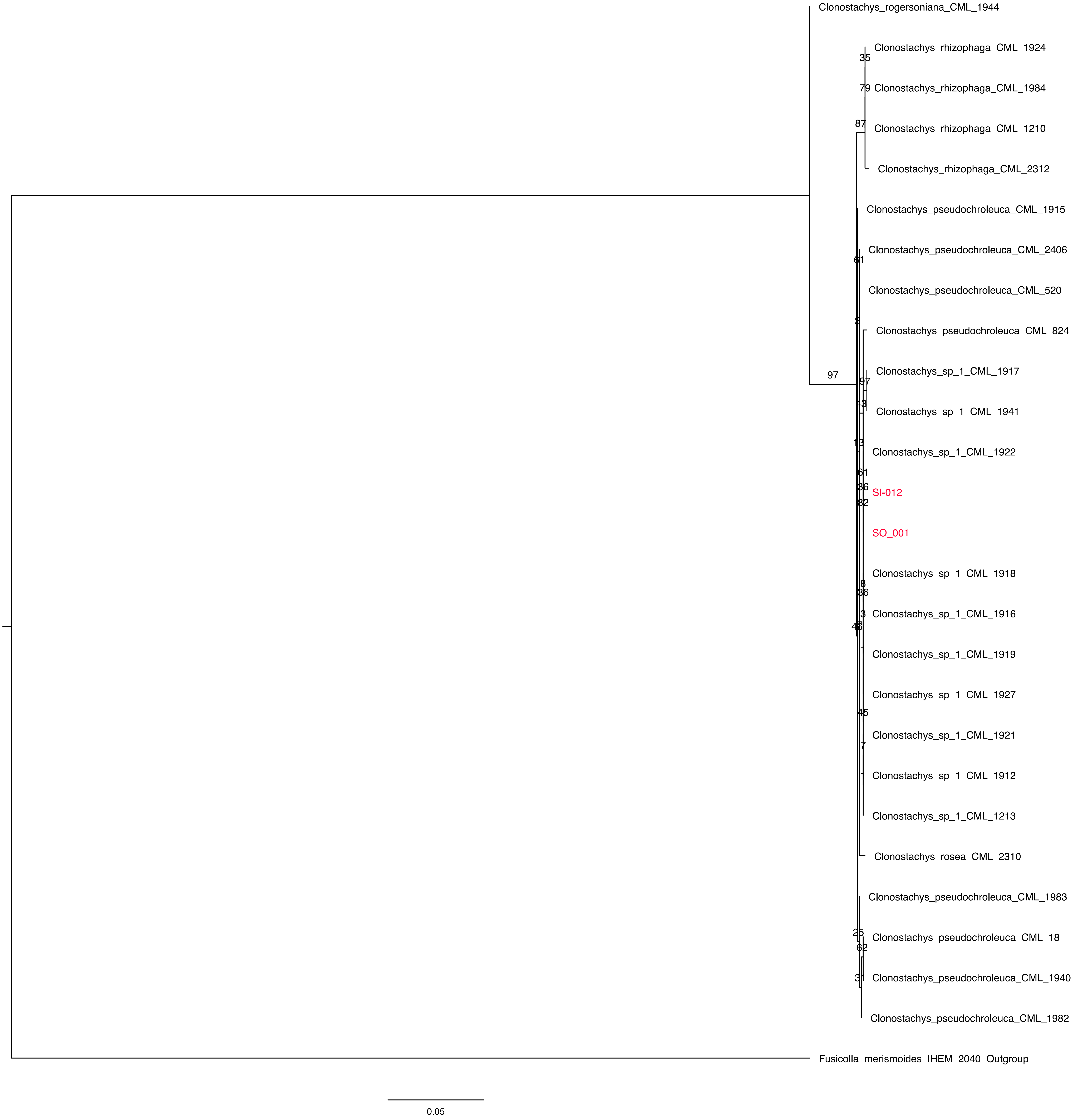
Clonostachys_rogersoniana_CML_1944
Clonostachys_rhizophaga_CML_1924
Clonostachys_rhizophaga_CML_1984
Clonostachys_rhizophaga_CML_1210
Clonostachys_rhizophaga_CML_2312
Clonostachys_pseudochroleuca_CML_1915
Clonostachys_pseudochroleuca_CML_2406
Clonostachys_pseudochroleuca_CML_520
Clonostachys_pseudochroleuca_CML_824
Clonostachys_sp_1_CML_1917
Clonostachys_sp_1_CML_1941
Clonostachys_sp_1_CML_1922
SI-012
SO_001
Clonostachys_sp_1_CML_1918
Clonostachys_sp_1_CML_1916
Clonostachys_sp_1_CML_1919
Clonostachys_sp_1_CML_1927
Clonostachys_sp_1_CML_1921
Clonostachys_sp_1_CML_1912
Clonostachys_sp_1_CML_1213
Clonostachys_rosea_CML_2310
Clonostachys_pseudochroleuca_CML_1983
Clonostachys_pseudochroleuca_CML_18
Clonostachys_pseudochroleuca_CML_1940
Clonostachys_pseudochroleuca_CML_1982
Fusicolla_merismoides_IHEM_2040_Outgroup
97
0.05

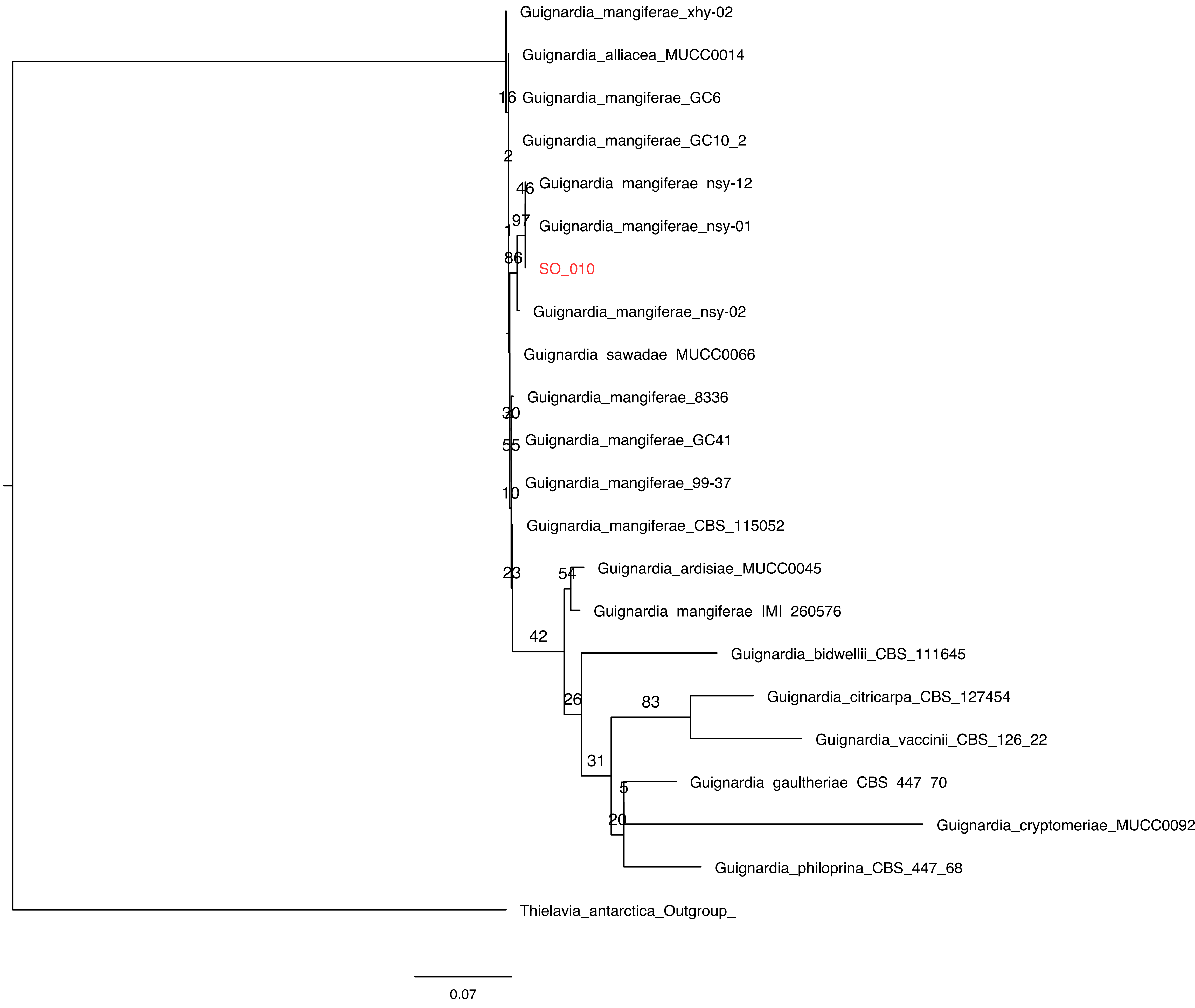
Guignardia_mangiferae_xhy-02
Guignardia_alliacea_MUCC0014
16
Guignardia_mangiferae_GC6
Guignardia_mangiferae_GC10_2
2
46
Guignardia_mangiferae_nsy-12
97
Guignardia_mangiferae_nsy-01
86
SO_010
Guignardia_mangiferae_nsy-02
Guignardia_sawadae_MUCC0066
Guignardia_mangiferae_8336
30
55
Guignardia_mangiferae_GC41
Guignardia_mangiferae_99-37
10
Guignardia_mangiferae_CBS_115052
23
54
Guignardia_ardisiae_MUCC0045
Guignardia_mangiferae_IMI_260576
42
Guignardia_bidwellii_CBS_111645
26
83
Guignardia_citricarpa_CBS_127454
Guignardia_vaccinii_CBS_126_22
31
5
Guignardia_gaultheriae_CBS_447_70
20
Guignardia_cryptomeriae_MUCC0092
Guignardia_philoprina_CBS_447_68
Thielavia_antarctica_Outgroup_
0.07

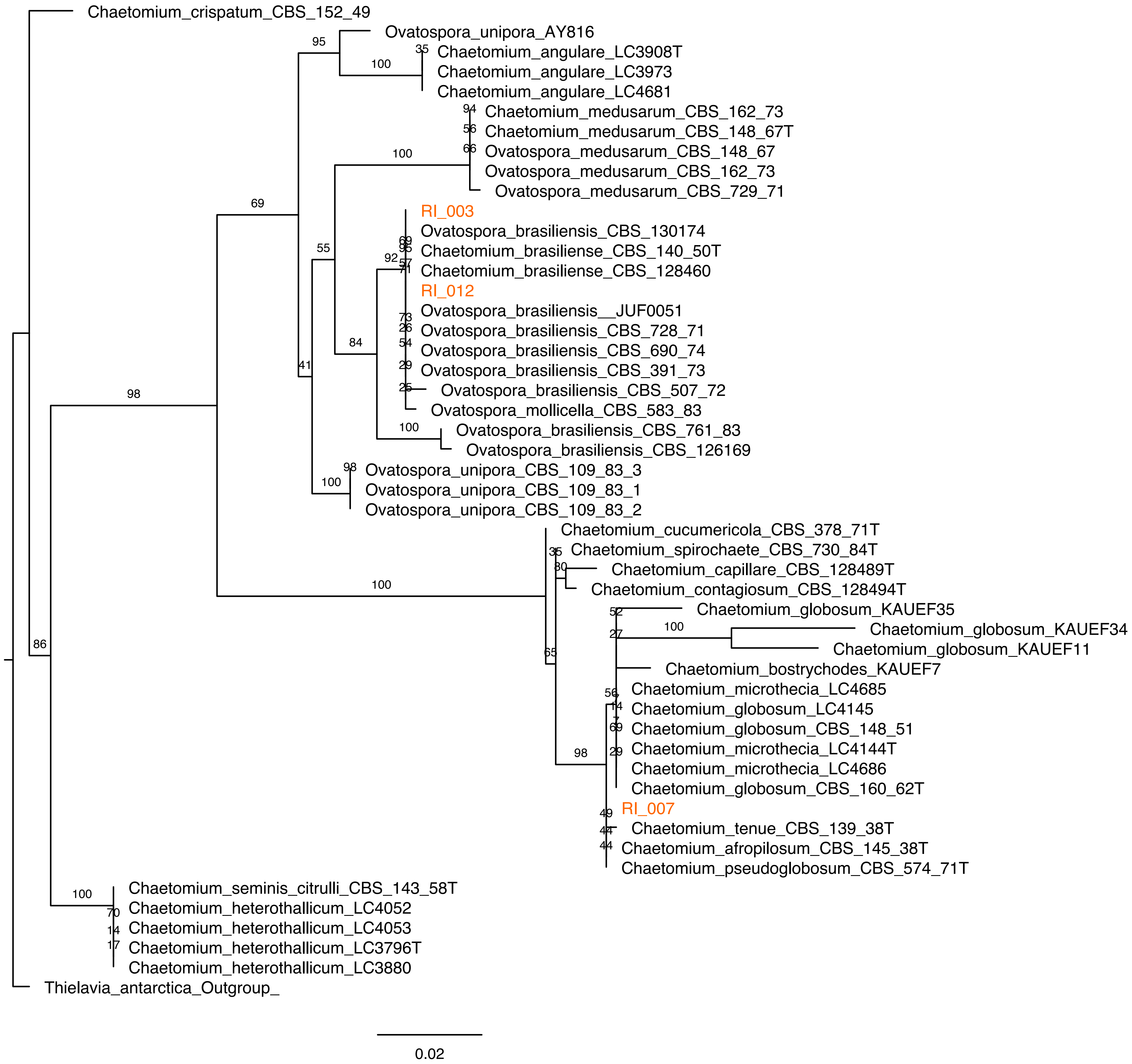
Chaetomium_crispatum_CBS_152_49
Ovatospora_unipora_AY816
Chaetomium_angulare_LC3908T
Chaetomium_angulare_LC3973
Chaetomium_angulare_LC4681
Chaetomium_medusarum_CBS_162_73
Chaetomium_medusarum_CBS_148_67T
Ovatospora_medusarum_CBS_148_67
Ovatospora_medusarum_CBS_162_73
Ovatospora_medusarum_CBS_729_71
RI_003
Ovatospora_brasiliensis_CBS_130174
Chaetomium_brasiliense_CBS_140_50T
Chaetomium_brasiliense_CBS_128460
RI_012
Ovatospora_brasiliensis__JUF0051
Ovatospora_brasiliensis_CBS_728_71
Ovatospora_brasiliensis_CBS_690_74
Ovatospora_brasiliensis_CBS_391_73
Ovatospora_brasiliensis_CBS_507_72
Ovatospora_mollicella_CBS_583_83
Ovatospora_brasiliensis_CBS_761_83
Ovatospora_brasiliensis_CBS_126169
Ovatospora_unipora_CBS_109_83_3
Ovatospora_unipora_CBS_109_83_1
Ovatospora_unipora_CBS_109_83_2
Chaetomium_cucumericola_CBS_378_71T
Chaetomium_spirochaete_CBS_730_84T
Chaetomium_capillare_CBS_128489T
Chaetomium_contagiosum_CBS_128494T
Chaetomium_globosum_KAUEF35
Chaetomium_globosum_KAUEF34
Chaetomium_globosum_KAUEF11
Chaetomium_bostrychodes_KAUEF7
Chaetomium_microthecia_LC4685
Chaetomium_globosum_LC4145
Chaetomium_globosum_CBS_148_51
Chaetomium_microthecia_LC4144T
Chaetomium_microthecia_LC4686
Chaetomium_globosum_CBS_160_62T
RI_007
Chaetomium_tenue_CBS_139_38T
Chaetomium_afropilosum_CBS_145_38T
Chaetomium_pseudoglobosum_CBS_574_71T
Chaetomium_seminis_citrulli_CBS_143_58T
Chaetomium_heterothallicum_LC4052
Chaetomium_heterothallicum_LC4053
Chaetomium_heterothallicum_LC3796T
Chaetomium_heterothallicum_LC3880
Thielavia_antarctica_Outgroup_
0.02

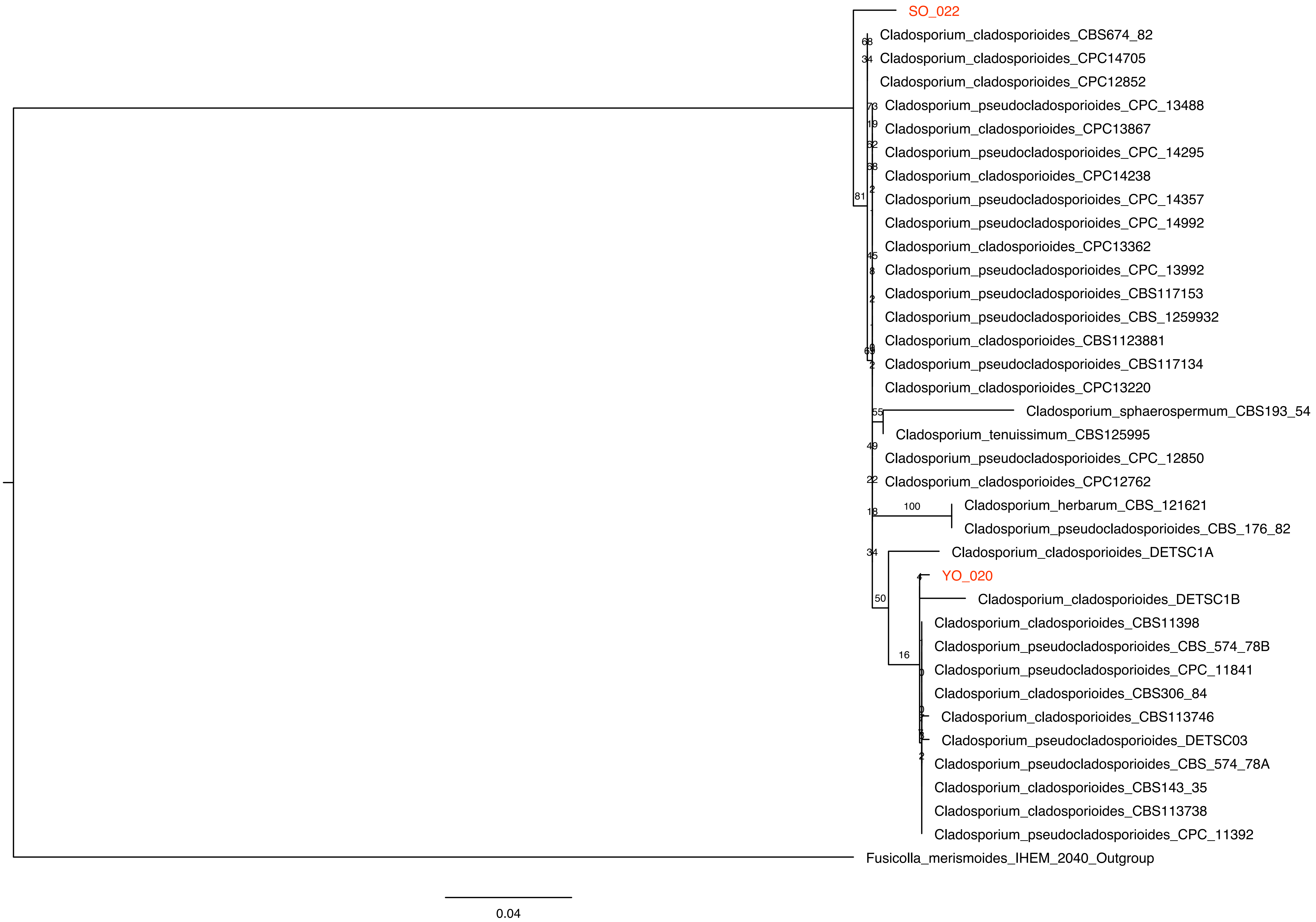
SO_022
Cladosporium_cladosporioides_CBS674_82
Cladosporium_cladosporioides_CPC14705
Cladosporium_cladosporioides_CPC12852
Cladosporium_pseudocladosporioides_CPC_13488
Cladosporium_cladosporioides_CPC13867
Cladosporium_pseudocladosporioides_CPC_14295
Cladosporium_cladosporioides_CPC14238
Cladosporium_pseudocladosporioides_CPC_14357
Cladosporium_pseudocladosporioides_CPC_14992
Cladosporium_cladosporioides_CPC13362
Cladosporium_pseudocladosporioides_CPC_13992
Cladosporium_pseudocladosporioides_CBS117153
Cladosporium_pseudocladosporioides_CBS_1259932
Cladosporium_cladosporioides_CBS1123881
Cladosporium_pseudocladosporioides_CBS117134
Cladosporium_cladosporioides_CPC13220
Cladosporium_sphaerospermum_CBS193_54
Cladosporium_tenuissimum_CBS125995
Cladosporium_pseudocladosporioides_CPC_12850
Cladosporium_cladosporioides_CPC12762
Cladosporium_herbarum_CBS_121621
Cladosporium_pseudocladosporioides_CBS_176_82
Cladosporium_cladosporioides_DETSC1A
YO_020
Cladosporium_cladosporioides_DETSC1B
Cladosporium_cladosporioides_CBS11398
Cladosporium_pseudocladosporioides_CBS_574_78B
Cladosporium_pseudocladosporioides_CPC_11841
Cladosporium_cladosporioides_CBS306_84
Cladosporium_cladosporioides_CBS113746
Cladosporium_pseudocladosporioides_DETSC03
Cladosporium_pseudocladosporioides_CBS_574_78A
Cladosporium_cladosporioides_CBS143_35
Cladosporium_cladosporioides_CBS113738
Cladosporium_pseudocladosporioides_CPC_11392
Fusicolla_merismoides_IHEM_2040_Outgroup
81
55
49
100
50
16
0.04

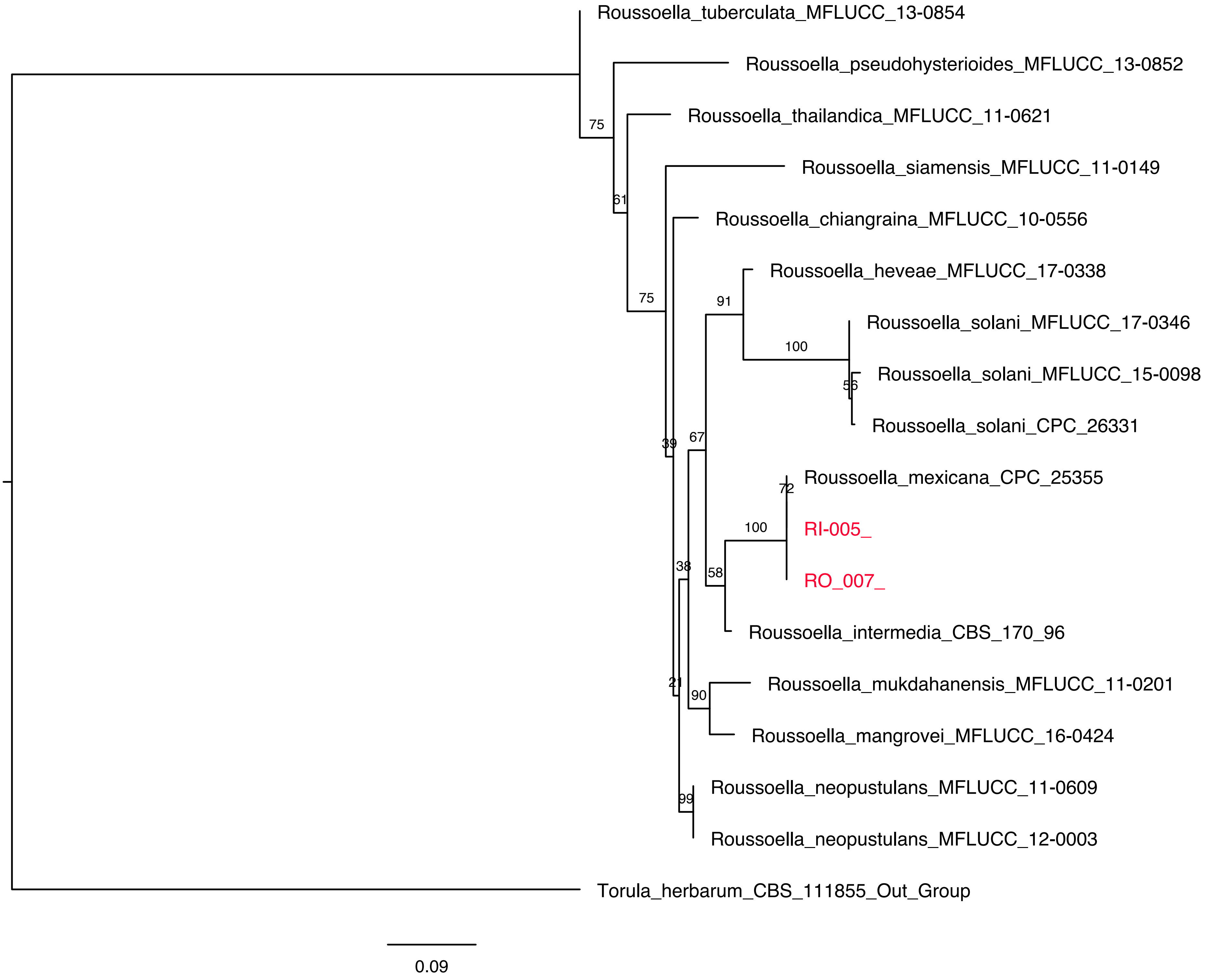
Roussoella_tuberculata_MFLUCC_13-0854
Roussoella_pseudohysterioides_MFLUCC_13-0852
Roussoella_thailandica_MFLUCC_11-0621
Roussoella_siamensis_MFLUCC_11-0149
Roussoella_chiangraina_MFLUCC_10-0556
Roussoella_heveae_MFLUCC_17-0338
Roussoella_solani_MFLUCC_17-0346
Roussoella_solani_MFLUCC_15-0098
Roussoella_solani_CPC_26331
Roussoella_mexicana_CPC_25355
RI-005_
RO_007_
Roussoella_intermedia_CBS_170_96
Roussoella_mukdahanensis_MFLUCC_11-0201
Roussoella_mangrovei_MFLUCC_16-0424
Roussoella_neopustulans_MFLUCC_11-0609
Roussoella_neopustulans_MFLUCC_12-0003
Torula_herbarum_CBS_111855_Out_Group
75
61
75
39
91
100
56
67
72
100
38
58
21
90
99
0.09

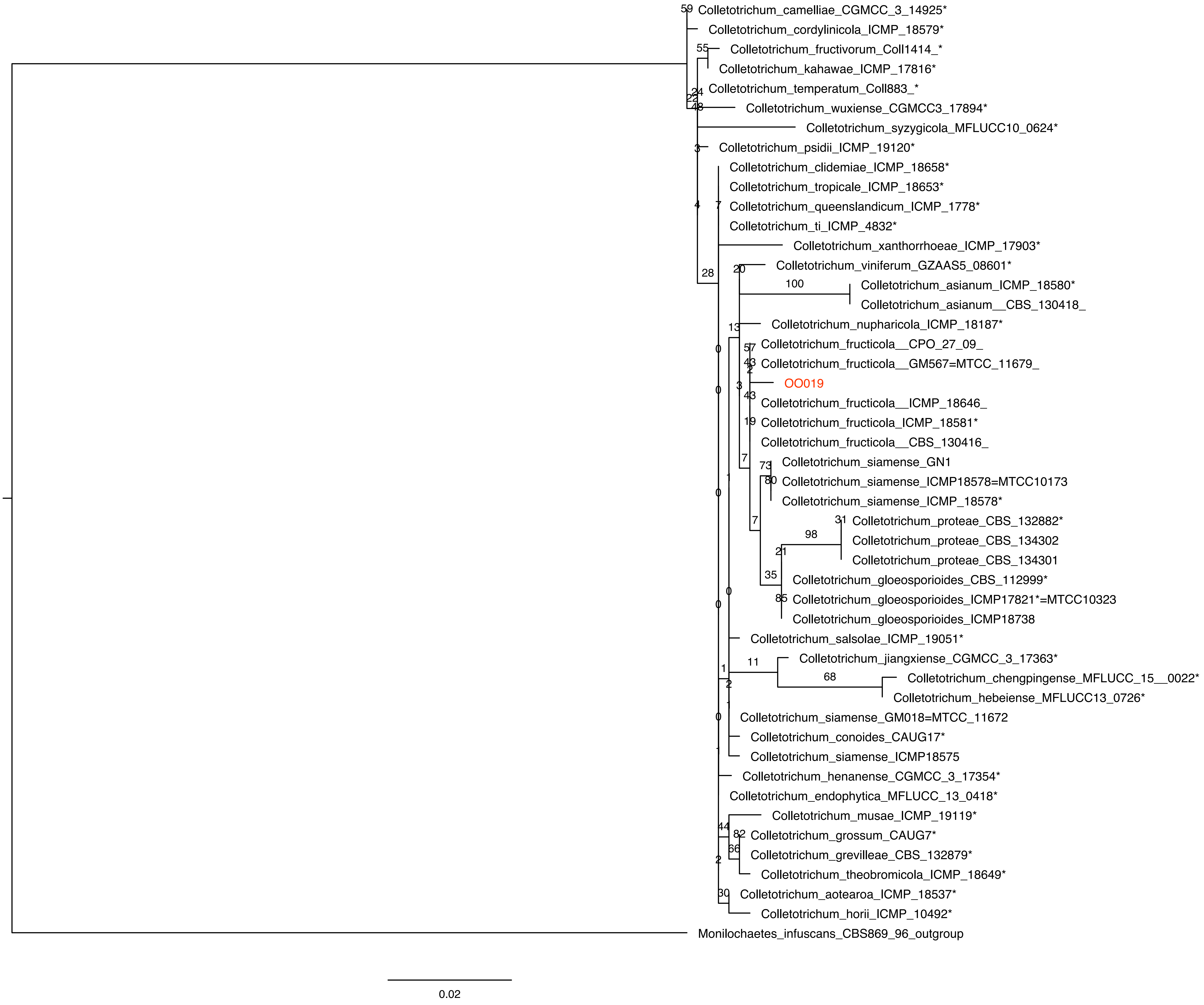
Colletotrichum_camelliae_CGMCC_3_14925*
Colletotrichum_cordylinicola_ICMP_18579*
Colletotrichum_fructivorum_Coll1414_*
Colletotrichum_kahawae_ICMP_17816*
Colletotrichum_temperatum_Coll883_*
Colletotrichum_wuxiense_CGMCC3_17894*
Colletotrichum_syzygicola_MFLUCC10_0624*
Colletotrichum_psidii_ICMP_19120*
Colletotrichum_clidemiae_ICMP_18658*
Colletotrichum_tropicale_ICMP_18653*
Colletotrichum_queenslandicum_ICMP_1778*
Colletotrichum_ti_ICMP_4832*
Colletotrichum_xanthorrhoeae_ICMP_17903*
Colletotrichum_viniferum_GZAAS5_08601*
Colletotrichum_asianum_ICMP_18580*
Colletotrichum_asianum__CBS_130418_
Colletotrichum_nupharicola_ICMP_18187*
Colletotrichum_fructicola__CPO_27_09_
Colletotrichum_fructicola__GM567=MTCC_11679_
OO019
Colletotrichum_fructicola__ICMP_18646_
Colletotrichum_fructicola_ICMP_18581*
Colletotrichum_fructicola__CBS_130416_
Colletotrichum_siamense_GN1
Colletotrichum_siamense_ICMP18578=MTCC10173
Colletotrichum_siamense_ICMP_18578*
Colletotrichum_proteae_CBS_132882*
Colletotrichum_proteae_CBS_134302
Colletotrichum_proteae_CBS_134301
Colletotrichum_gloeosporioides_CBS_112999*
Colletotrichum_gloeosporioides_ICMP17821*=MTCC10323
Colletotrichum_gloeosporioides_ICMP18738
Colletotrichum_salsolae_ICMP_19051*
Colletotrichum_jiangxiense_CGMCC_3_17363*
Colletotrichum_chengpingense_MFLUCC_15__0022*
Colletotrichum_hebeiense_MFLUCC13_0726*
Colletotrichum_siamense_GM018=MTCC_11672
Colletotrichum_conoides_CAUG17*
Colletotrichum_siamense_ICMP18575
Colletotrichum_henanense_CGMCC_3_17354*
Colletotrichum_endophytica_MFLUCC_13_0418*
Colletotrichum_musae_ICMP_19119*
Colletotrichum_grossum_CAUG7*
Colletotrichum_grevilleae_CBS_132879*
Colletotrichum_theobromicola_ICMP_18649*
Colletotrichum_aotearoa_ICMP_18537*
Colletotrichum_horii_ICMP_10492*
Monilochaetes_infuscans_CBS869_96_outgroup
0.02

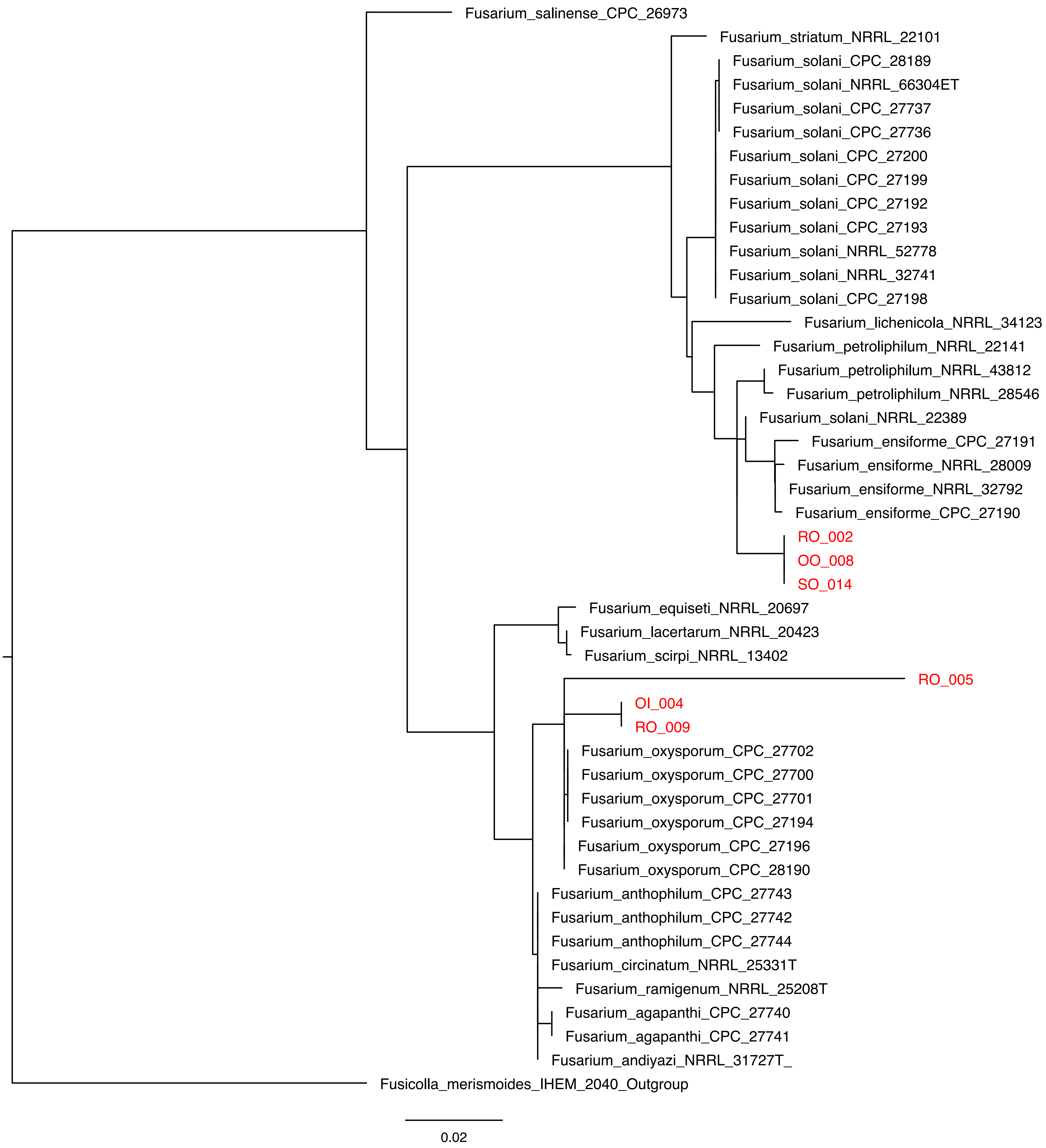
Fusarium_salinense_CPC_26973
Fusarium_striatum_NRRL_22101
Fusarium_solani_CPC_28189
Fusarium_solani_NRRL_66304ET
Fusarium_solani_CPC_27737
Fusarium_solani_CPC_27736
Fusarium_solani_CPC_27200
Fusarium_solani_CPC_27199
Fusarium_solani_CPC_27192
Fusarium_solani_CPC_27193
Fusarium_solani_NRRL_52778
Fusarium_solani_NRRL_32741
Fusarium_solani_CPC_27198
Fusarium_lichenicola_NRRL_34123
Fusarium_petroliphilum_NRRL_22141
Fusarium_petroliphilum_NRRL_43812
Fusarium_petroliphilum_NRRL_28546
Fusarium_solani_NRRL_22389
Fusarium_ensiforme_CPC_27191
Fusarium_ensiforme_NRRL_28009
Fusarium_ensiforme_NRRL_32792
Fusarium_ensiforme_CPC_27190
RO_002
OO_008
SO_014
Fusarium_equiseti_NRRL_20697
Fusarium_lacertarum_NRRL_20423
Fusarium_scirpi_NRRL_13402
RO_005
OI_004
RO_009
Fusarium_oxysporum_CPC_27702
Fusarium_oxysporum_CPC_27700
Fusarium_oxysporum_CPC_27701
Fusarium_oxysporum_CPC_27194
Fusarium_oxysporum_CPC_27196
Fusarium_oxysporum_CPC_28190
Fusarium_anthophilum_CPC_27743
Fusarium_anthophilum_CPC_27742
Fusarium_anthophilum_CPC_27744
Fusarium_circinatum_NRRL_25331T
Fusarium_ramigenum_NRRL_25208T
Fusarium_agapanthi_CPC_27740
Fusarium_agapanthi_CPC_27741
Fusarium_andiyazi_NRRL_31727T_
Fusicolla_merismoides_IHEM_2040_Outgroup
0.02

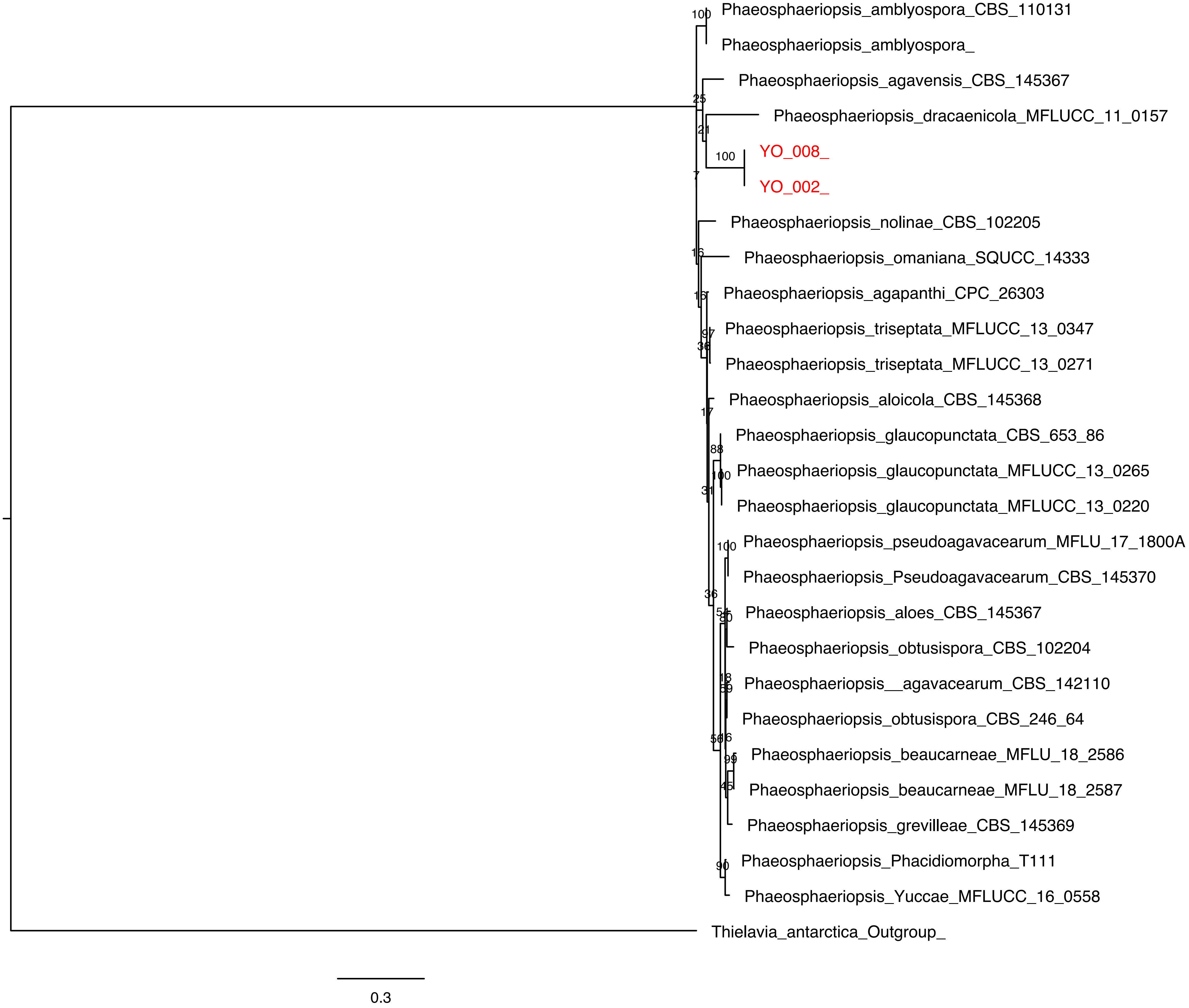
Phaeosphaeriopsis_amblyospora_CBS_110131
Phaeosphaeriopsis_amblyospora_
Phaeosphaeriopsis_agavensis_CBS_145367
Phaeosphaeriopsis_dracaenicola_MFLUCC_11_0157
YO_008_
YO_002_
Phaeosphaeriopsis_nolinae_CBS_102205
Phaeosphaeriopsis_omaniana_SQUCC_14333
Phaeosphaeriopsis_agapanthi_CPC_26303
Phaeosphaeriopsis_triseptata_MFLUCC_13_0347
Phaeosphaeriopsis_triseptata_MFLUCC_13_0271
Phaeosphaeriopsis_aloicola_CBS_145368
Phaeosphaeriopsis_glaucopunctata_CBS_653_86
Phaeosphaeriopsis_glaucopunctata_MFLUCC_13_0265
Phaeosphaeriopsis_glaucopunctata_MFLUCC_13_0220
Phaeosphaeriopsis_pseudoagavacearum_MFLU_17_1800A
Phaeosphaeriopsis_Pseudoagavacearum_CBS_145370
Phaeosphaeriopsis_aloes_CBS_145367
Phaeosphaeriopsis_obtusispora_CBS_102204
Phaeosphaeriopsis__agavacearum_CBS_142110
Phaeosphaeriopsis_obtusispora_CBS_246_64
Phaeosphaeriopsis_beaucarneae_MFLU_18_2586
Phaeosphaeriopsis_beaucarneae_MFLU_18_2587
Phaeosphaeriopsis_grevilleae_CBS_145369
Phaeosphaeriopsis_Phacidiomorpha_T111
Phaeosphaeriopsis_Yuccae_MFLUCC_16_0558
Thielavia_antarctica_Outgroup_
0.3

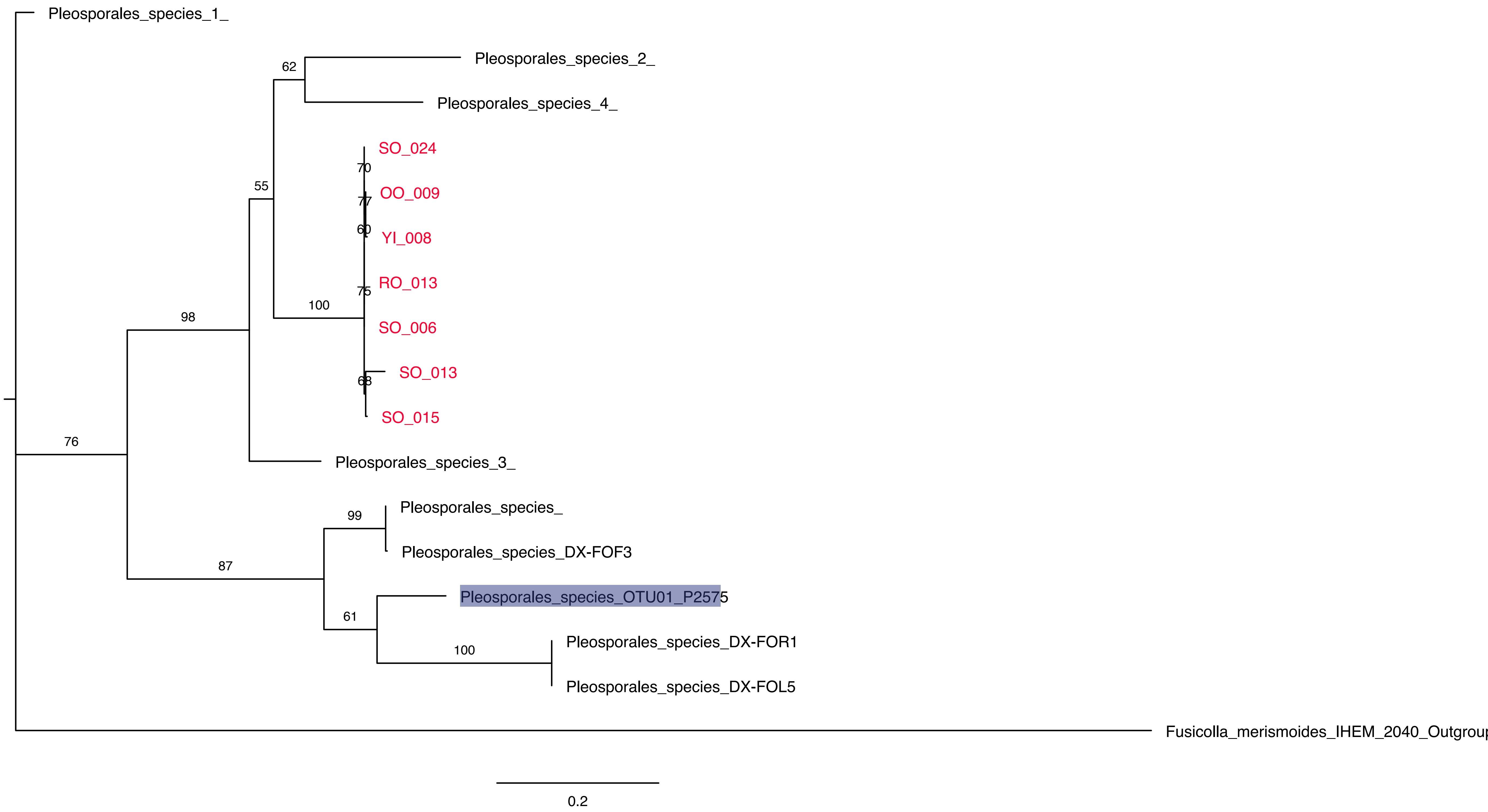
Pleosporales_species_1_
62
Pleosporales_species_2_
Pleosporales_species_4_
SO_024
70
55
OO_009
77
60
YI_008
RO_013
75
98
100
SO_006
SO_013
68
SO_015
76
Pleosporales_species_3_
Pleosporales_species_
99
Pleosporales_species_DX-FOF3
87
Pleosporales_species_OTU01_P2575
61
100
Pleosporales_species_DX-FOR1
Pleosporales_species_DX-FOL5
Fusicolla_merismoides_IHEM_2040_Outgrou
0.2

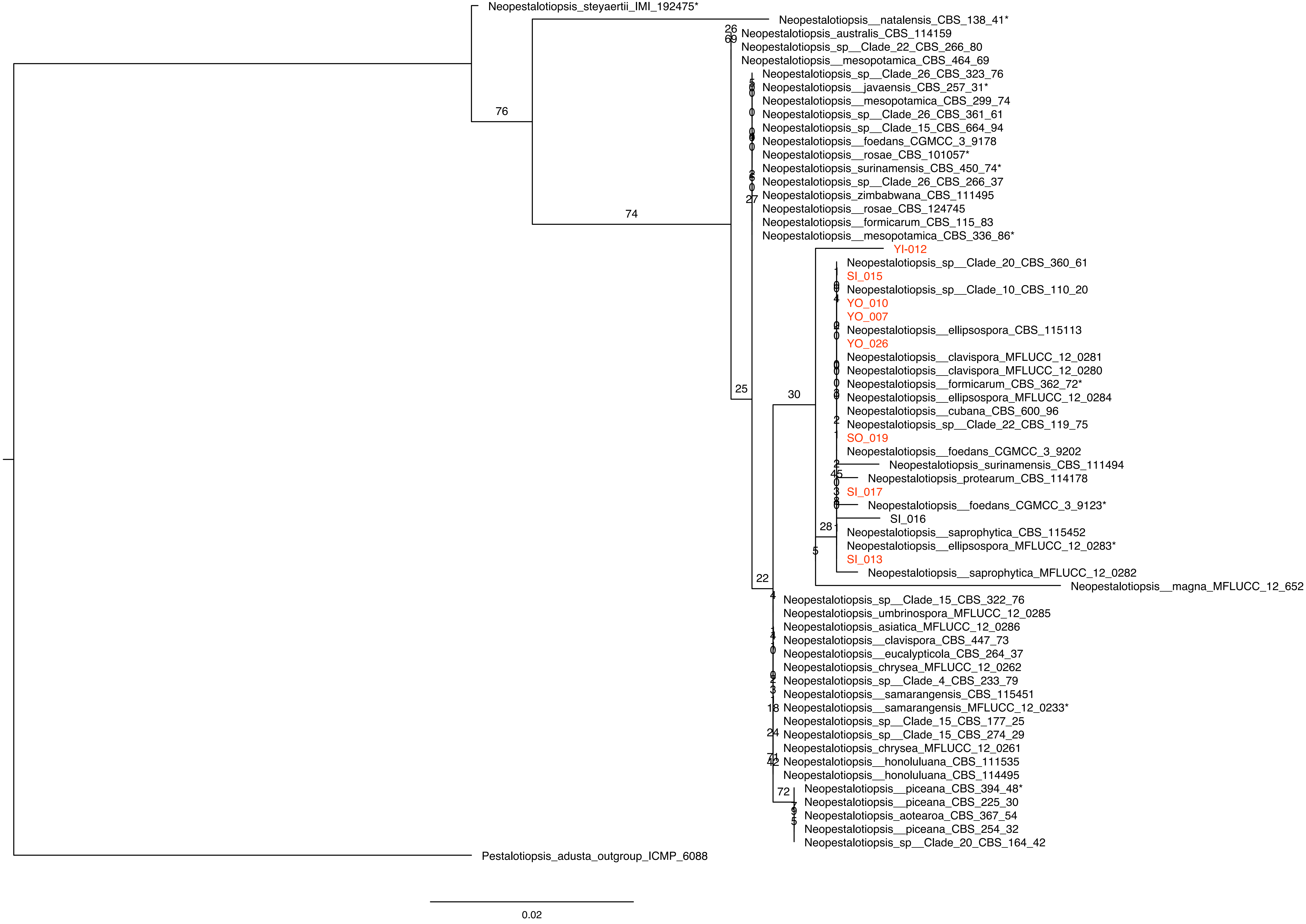

Neopestalotiopsis_steyaertii_IMI_192475*
Neopestalotiopsis__natalensis_CBS_138_41*
Neopestalotiopsis_australis_CBS_114159
Neopestalotiopsis_sp__Clade_22_CBS_266_80
Neopestalotiopsis__mesopotamica_CBS_464_69
Neopestalotiopsis_sp__Clade_26_CBS_323_76
Neopestalotiopsis__javaensis_CBS_257_31*
Neopestalotiopsis__mesopotamica_CBS_299_74
Neopestalotiopsis_sp__Clade_26_CBS_361_61
Neopestalotiopsis_sp__Clade_15_CBS_664_94
Neopestalotiopsis__foedans_CGMCC_3_9178
Neopestalotiopsis__rosae_CBS_101057*
Neopestalotiopsis_surinamensis_CBS_450_74*
Neopestalotiopsis_sp__Clade_26_CBS_266_37
Neopestalotiopsis_zimbabwana_CBS_111495
Neopestalotiopsis__rosae_CBS_124745
Neopestalotiopsis__formicarum_CBS_115_83
Neopestalotiopsis__mesopotamica_CBS_336_86*
YI-012
Neopestalotiopsis_sp__Clade_20_CBS_360_61
SI_015
Neopestalotiopsis_sp__Clade_10_CBS_110_20
YO_010
YO_007
Neopestalotiopsis__ellipsospora_CBS_115113
YO_026
Neopestalotiopsis__clavispora_MFLUCC_12_0281
Neopestalotiopsis__clavispora_MFLUCC_12_0280
Neopestalotiopsis__formicarum_CBS_362_72*
Neopestalotiopsis__ellipsospora_MFLUCC_12_0284
Neopestalotiopsis__cubana_CBS_600_96
Neopestalotiopsis_sp__Clade_22_CBS_119_75
SO_019
Neopestalotiopsis__foedans_CGMCC_3_9202
Neopestalotiopsis__surinamensis_CBS_111494
Neopestalotiopsis__protearum_CBS_114178
SI_017
Neopestalotiopsis__foedans_CGMCC_3_9123*
SI_016
Neopestalotiopsis__saprophytica_CBS_115452
Neopestalotiopsis__ellipsospora_MFLUCC_12_0283*
SI_013
Neopestalotiopsis__saprophytica_MFLUCC_12_0282
Neopestalotiopsis__magna_MFLUCC_12_652
Neopestalotiopsis_sp__Clade_15_CBS_322_76
Neopestalotiopsis_umbrinospora_MFLUCC_12_0285
Neopestalotiopsis_asiatica_MFLUCC_12_0286
Neopestalotiopsis__clavispora_CBS_447_73
Neopestalotiopsis__eucalypticola_CBS_264_37
Neopestalotiopsis_chrysea_MFLUCC_12_0262
Neopestalotiopsis_sp__Clade_4_CBS_233_79
Neopestalotiopsis__samarangensis_CBS_115451
Neopestalotiopsis__samarangensis_MFLUCC_12_0233*
Neopestalotiopsis_sp__Clade_15_CBS_177_25
Neopestalotiopsis_sp__Clade_15_CBS_274_29
Neopestalotiopsis_chrysea_MFLUCC_12_0261
Neopestalotiopsis__honoluluana_CBS_111535
Neopestalotiopsis__honoluluana_CBS_114495
Neopestalotiopsis__piceana_CBS_394_48*
Neopestalotiopsis__piceana_CBS_225_30
Neopestalotiopsis_aotearoa_CBS_367_54
Neopestalotiopsis__piceana_CBS_254_32
Neopestalotiopsis_sp__Clade_20_CBS_164_42
Pestalotiopsis_adusta_outgroup_ICMP_6088
76
74
26
69
25
30
28
22
72
0.02

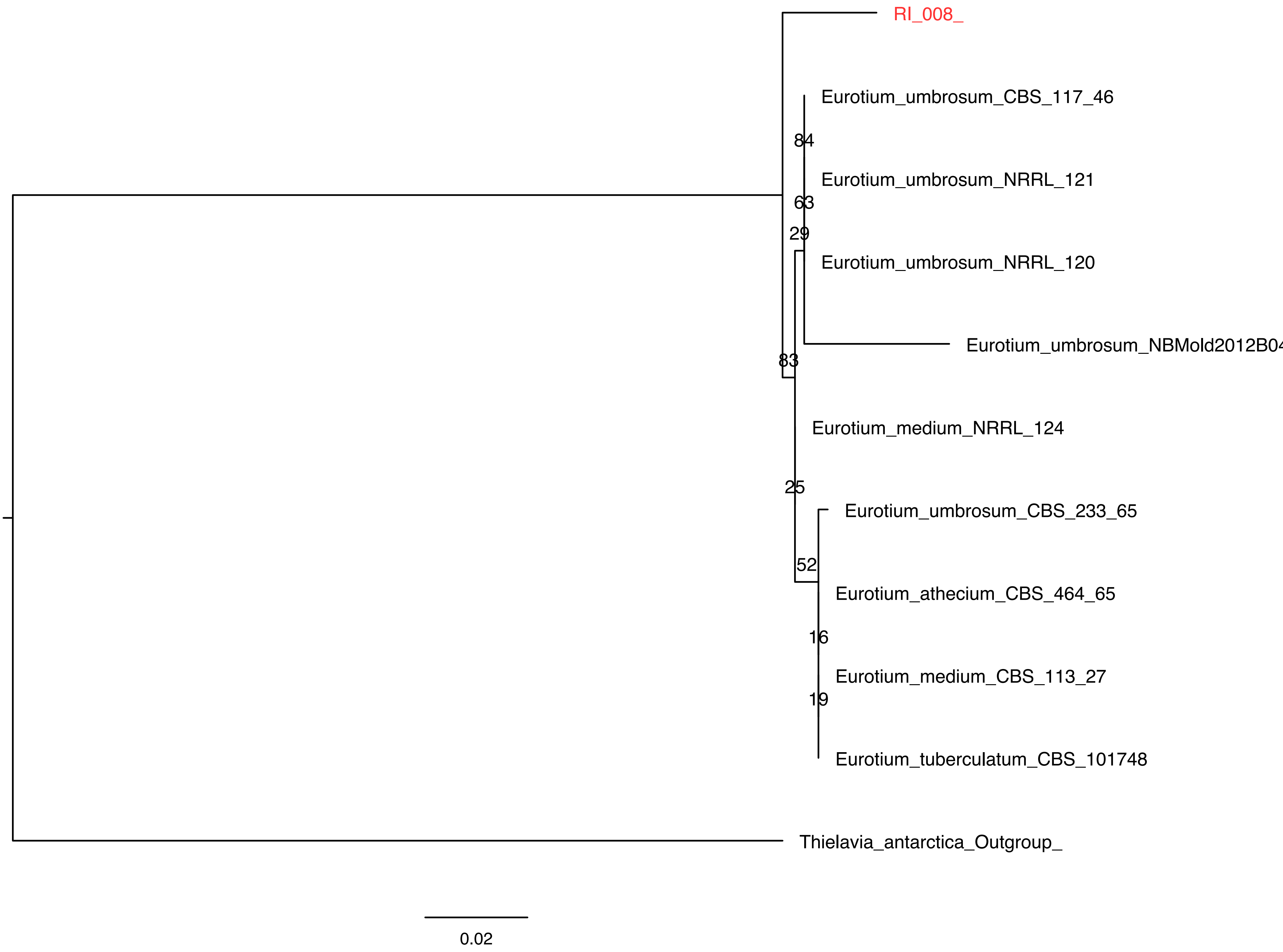
RI_008_
Eurotium_umbrosum_CBS_117_46
84
Eurotium_umbrosum_NRRL_121
63
29
Eurotium_umbrosum_NRRL_120
Eurotium_umbrosum_NBMold2012B04
83
Eurotium_medium_NRRL_124
25
Eurotium_umbrosum_CBS_233_65
52
Eurotium_athecium_CBS_464_65
16
Eurotium_medium_CBS_113_27
19
Eurotium_tuberculatum_CBS_101748
Thielavia_antarctica_Outgroup_
0.02

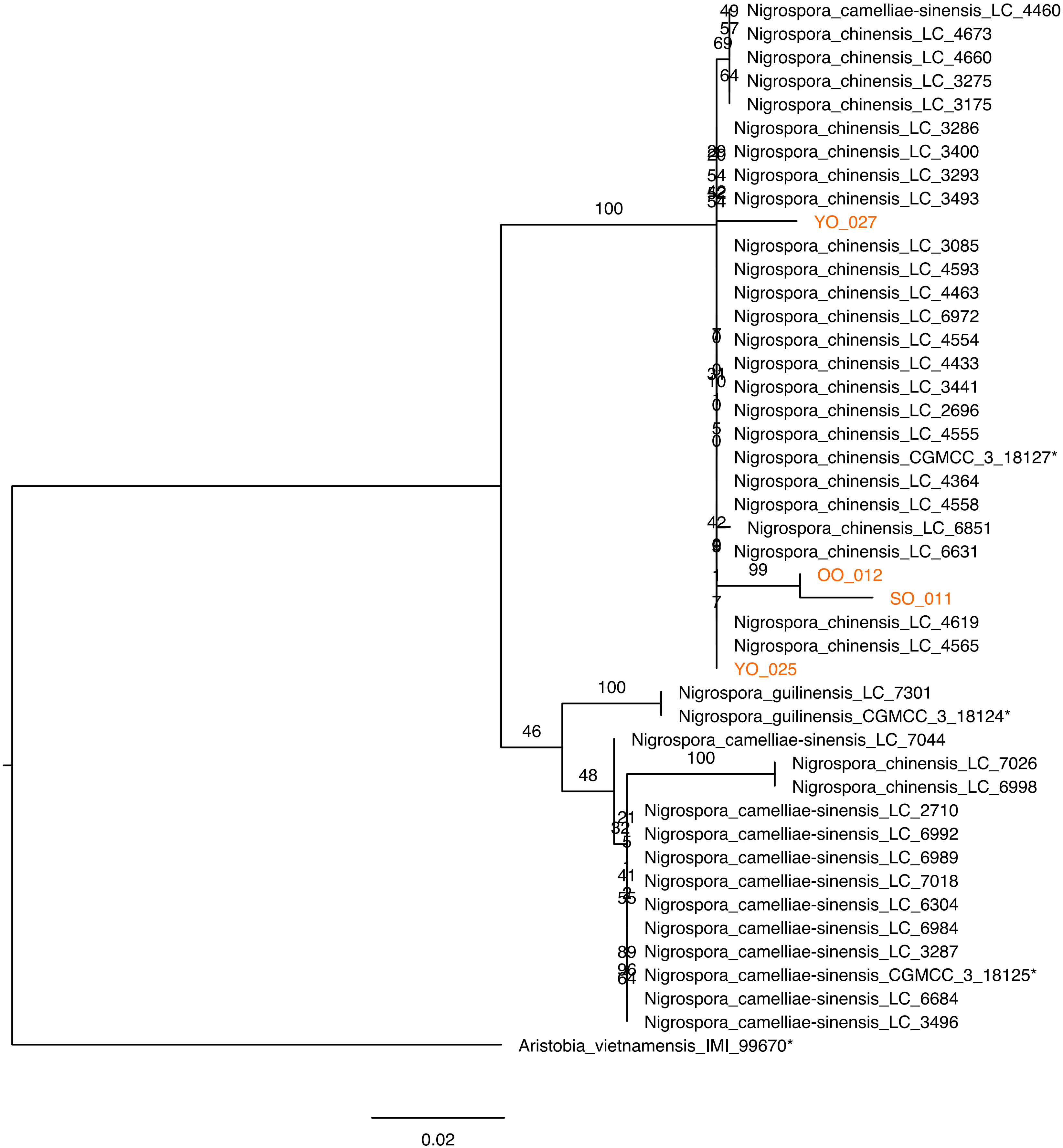
Nigrospora_camelliae-sinensis_LC_4460
Nigrospora_chinensis_LC_4673
Nigrospora_chinensis_LC_4660
Nigrospora_chinensis_LC_3275
Nigrospora_chinensis_LC_3175
Nigrospora_chinensis_LC_3286
Nigrospora_chinensis_LC_3400
Nigrospora_chinensis_LC_3293
Nigrospora_chinensis_LC_3493
YO_027
Nigrospora_chinensis_LC_3085
Nigrospora_chinensis_LC_4593
Nigrospora_chinensis_LC_4463
Nigrospora_chinensis_LC_6972
Nigrospora_chinensis_LC_4554
Nigrospora_chinensis_LC_4433
Nigrospora_chinensis_LC_3441
Nigrospora_chinensis_LC_2696
Nigrospora_chinensis_LC_4555
Nigrospora_chinensis_CGMCC_3_18127*
Nigrospora_chinensis_LC_4364
Nigrospora_chinensis_LC_4558
Nigrospora_chinensis_LC_6851
Nigrospora_chinensis_LC_6631
OO_012
SO_011
Nigrospora_chinensis_LC_4619
Nigrospora_chinensis_LC_4565
YO_025
Nigrospora_guilinensis_LC_7301
Nigrospora_guilinensis_CGMCC_3_18124*
Nigrospora_camelliae-sinensis_LC_7044
Nigrospora_chinensis_LC_7026
Nigrospora_chinensis_LC_6998
Nigrospora_camelliae-sinensis_LC_2710
Nigrospora_camelliae-sinensis_LC_6992
Nigrospora_camelliae-sinensis_LC_6989
Nigrospora_camelliae-sinensis_LC_7018
Nigrospora_camelliae-sinensis_LC_6304
Nigrospora_camelliae-sinensis_LC_6984
Nigrospora_camelliae-sinensis_LC_3287
Nigrospora_camelliae-sinensis_CGMCC_3_18125*
Nigrospora_camelliae-sinensis_LC_6684
Nigrospora_camelliae-sinensis_LC_3496
Aristobia_vietnamensis_IMI_99670*
100
99
100
46
48
100
0.02

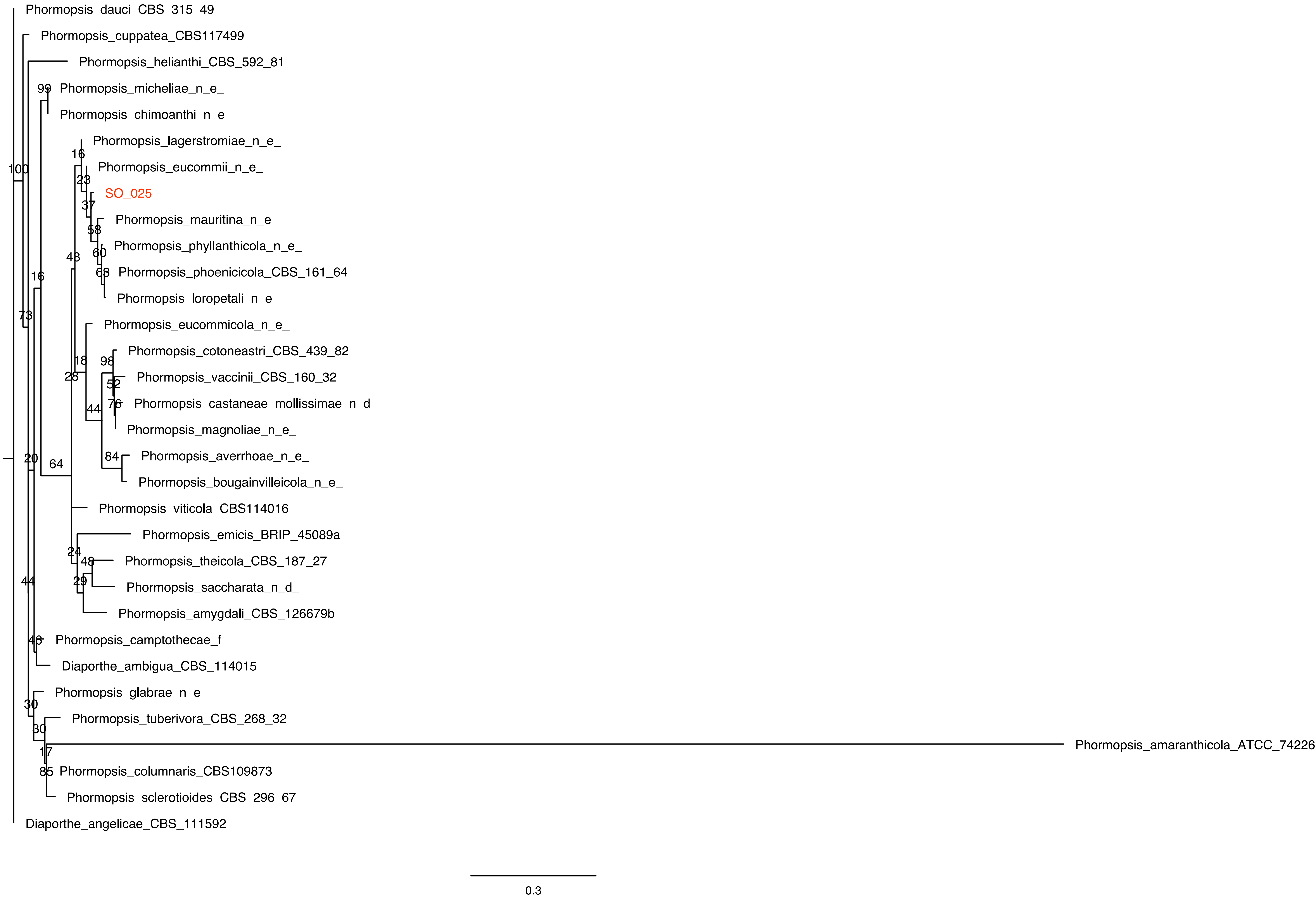
Phormopsis_dauci_CBS_315_49
Phormopsis_cuppatea_CBS117499
Phormopsis_helianthi_CBS_592_81
Phormopsis_micheliae_n_e_
Phormopsis_chimoanthi_n_e
Phormopsis_lagerstromiae_n_e_
Phormopsis_eucommii_n_e_
SO_025
Phormopsis_mauritina_n_e
Phormopsis_phyllanthicola_n_e_
Phormopsis_phoenicicola_CBS_161_64
Phormopsis_loropetali_n_e_
Phormopsis_eucommicola_n_e_
Phormopsis_cotoneastri_CBS_439_82
Phormopsis_vaccinii_CBS_160_32
Phormopsis_castaneae_mollissimae_n_d_
Phormopsis_magnoliae_n_e_
Phormopsis_averrhoae_n_e_
Phormopsis_bougainvilleicola_n_e_
Phormopsis_viticola_CBS114016
Phormopsis_emicis_BRIP_45089a
Phormopsis_theicola_CBS_187_27
Phormopsis_saccharata_n_d_
Phormopsis_amygdali_CBS_126679b
Phormopsis_camptothecae_f
Diaporthe_ambigua_CBS_114015
Phormopsis_glabrae_n_e
Phormopsis_tuberivora_CBS_268_32
Phormopsis_amaranthicola_ATCC_74226
Phormopsis_columnaris_CBS109873
Phormopsis_sclerotioides_CBS_296_67
Diaporthe_angelicae_CBS_111592
0.3

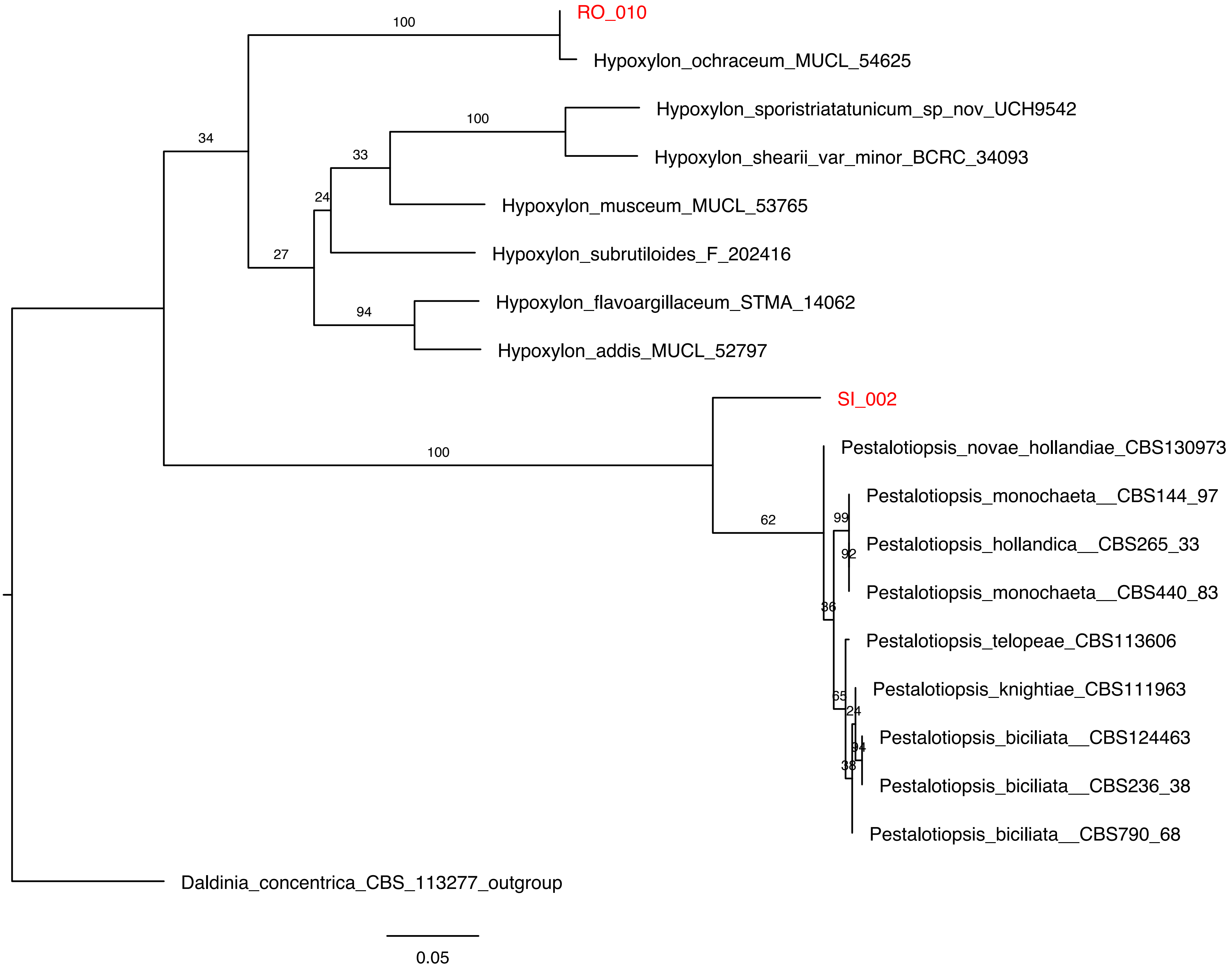
RO_010
Hypoxylon_ochraceum_MUCL_54625
Hypoxylon_sporistriatatunicum_sp_nov_UCH9542
Hypoxylon_shearii_var_minor_BCRC_34093
Hypoxylon_musceum_MUCL_53765
Hypoxylon_subrutiloides_F_202416
Hypoxylon_flavoargillaceum_STMA_14062
Hypoxylon_addis_MUCL_52797
SI_002
Pestalotiopsis_novae_hollandiae_CBS130973
Pestalotiopsis_monochaeta__CBS144_97
Pestalotiopsis_hollandica__CBS265_33
Pestalotiopsis_monochaeta__CBS440_83
Pestalotiopsis_telopeae_CBS113606
Pestalotiopsis_knightiae_CBS111963
Pestalotiopsis_biciliata__CBS124463
Pestalotiopsis_biciliata__CBS236_38
Pestalotiopsis_biciliata__CBS790_68
Daldinia_concentrica_CBS_113277_outgroup
100
34
100
33
24
27
94
100
62
99
92
36
65
24
94
38
0.05

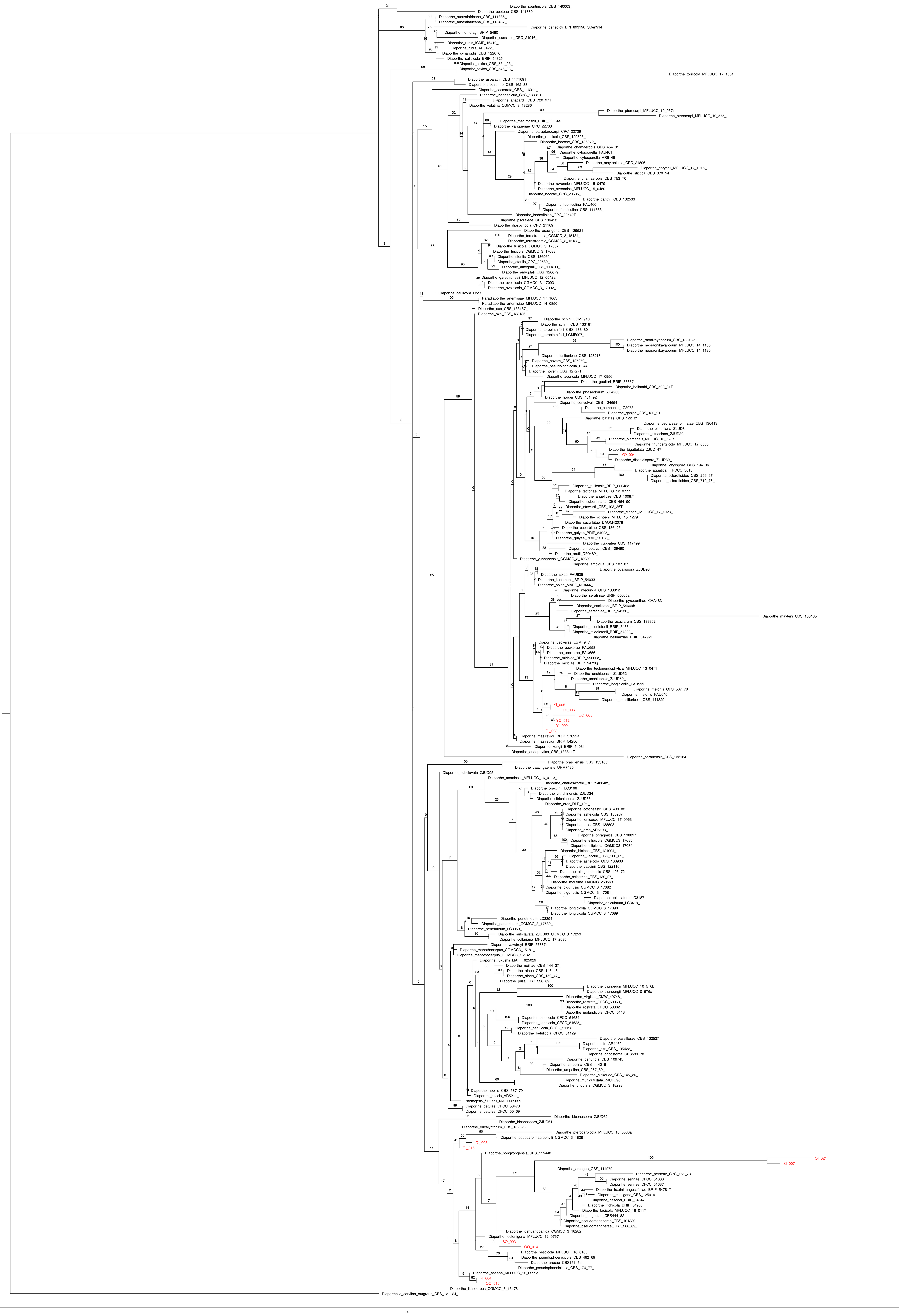
Diaporthe_spartinicola_CBS_140003_
Diaporthe_ocoteae_CBS_141330
Diaporthe_australafricana_CBS_111886_
Diaporthe_australafricana_CBS_113487_
Diaporthe_benedicti_BPI_893190_SBen914
Diaporthe_nothofagi_BRIP_54801_
Diaporthe_cassines_CPC_21916_
Diaporthe_rudis_ICMP_16419_
Diaporthe_rudis_AR3422_
Diaporthe_cynaroidis_CBS_122676_
Diaporthe_salicicola_BRIP_54825_
Diaporthe_toxica_CBS_534_93_
Diaporthe_toxica_CBS_546_93_
Diaporthe_torilicola_MFLUCC_17_1051
Diaporthe_aspalathi_CBS_117169T
Diaporthe_crotalariae_CBS_162_33
Diaporthe_saccarata_CBS_116311_
Diaporthe_inconspicua_CBS_133813
Diaporthe_anacardii_CBS_720_97T
Diaporthe_velutina_CGMCC_3_18286
Diaporthe_pterocarpi_MFLUCC_10_0571
Diaporthe_pterocarpi_MFLUCC_10_575_
Diaporthe_macintoshii_BRIP_55064a
Diaporthe_vangueriae_CPC_22703
Diaporthe_parapterocarpi_CPC_22729
Diaporthe_rhusicola_CBS_129528_
Diaporthe_baccae_CBS_136972_
Diaporthe_chamaeropis_CBS_454_81_
Diaporthe_cytosporella_FAU461_
Diaporthe_cytosporella_AR5149_
Diaporthe_maytenicola_CPC_21896
Diaporthe_dorycnii_MFLUCC_17_1015_
Diaporthe_stictica_CBS_370_54
Diaporthe_chamaeropis_CBS_753_70_
Diaporthe_ravennica_MFLUCC_15_0479
Diaporthe_ravennica_MFLUCC_15_0480
Diaporthe_baccae_CPC_20585_
Diaporthe_canthii_CBS_132533_
Diaporthe_foeniculina_FAU460_
Diaporthe_foeniculina_CBS_111553_
Diaporthe_isoberliniae_CPC_22549T
Diaporthe_psoraleae_CBS_136412
Diaporthe_diospyricola_CPC_21169_
Diaporthe_acaciigena_CBS_129521_
Diaporthe_ternstroemia_CGMCC_3_15184_
Diaporthe_ternstroemia_CGMCC_3_15183_
Diaporthe_fusicola_CGMCC_3_17087_
Diaporthe_fusicola_CGMCC_3_17088_
Diaporthe_sterilis_CBS_136969_
Diaporthe_sterilis_CPC_20580_
Diaporthe_amygdali_CBS_111811_
Diaporthe_amygdali_CBS_126679_
Diaporthe_garethjonesii_MFLUCC_12_0542a
Diaporthe_ovoicicola_CGMCC_3_17093_
Diaporthe_ovoicicola_CGMCC_3_17092_
Diaporthe_caulivora_Dpc1
Paradiaporthe_artemisiae_MFLUCC_17_1663
Paradiaporthe_artemisiae_MFLUCC_14_0850
Diaporthe_oxe_CBS_133187_
Diaporthe_oxe_CBS_133186
Diaporthe_schini_LGMF910_
Diaporthe_schini_CBS_133181
Diaporthe_terebinthifolii_CBS_133180
Diaporthe_terebinthifolii_LGMF907_
Diaporthe_raonikayaporum_CBS_133182
Diaporthe_neoraonikayaporum_MFLUCC_14_1133_
Diaporthe_neoraonikayaporum_MFLUCC_14_1136_
Diaporthe_lusitanicae_CBS_123213
Diaporthe_novem_CBS_127270_
Diaporthe_pseudolongicolla_PL44
Diaporthe_novem_CBS_127271_
Diaporthe_acericola_MFLUCC_17_0956_
Diaporthe_goulteri_BRIP_55657a
Diaporthe_helianthi_CBS_592_81T
Diaporthe_phaseolorum_AR4203
Diaporthe_hordei_CBS_481_92
Diaporthe_convolvuli_CBS_124654
Diaporthe_compacta_LC3078
Diaporthe_ganjae_CBS_180_91
Diaporthe_batatas_CBS_122_21
Diaporthe_psoraleae_pinnatae_CBS_136413
Diaporthe_citriasiana_ZJUD81
Diaporthe_citriasiana_ZJUD30
Diaporthe_siamensis_MFLUCC10_573a
Diaporthe_thunbergiicola_MFLUCC_12_0033
Diaporthe_biguttulata_ZJUD_47
YO_004
Diaporthe_discoidispora_ZJUD89_
Diaporthe_longispora_CBS_194_36
Diaporthe_aquatica_IFRDCC_3015
Diaporthe_sclerotioides_CBS_296_67
Diaporthe_sclerotioides_CBS_710_76_
Diaporthe_tulliensis_BRIP_62248a
Diaporthe_tectonae_MFLUCC_12_0777
Diaporthe_angelicae_CBS_100871
Diaporthe_subordinaria_CBS_464_90
Diaporthe_stewartii_CBS_193_36T
Diaporthe_cichorii_MFLUCC_17_1023_
Diaporthe_schoeni_MFLU_15_1279
Diaporthe_cucurbitae_DAOM42078_
Diaporthe_cucurbitae_CBS_136_25_
Diaporthe_gulyae_BRIP_54025_
Diaporthe_gulyae_BRIP_53158_
Diaporthe_cuppatea_CBS_117499
Diaporthe_neoarctii_CBS_109490_
Diaporthe_arctii_DP0482_
Diaporthe_yunnanensis_CGMCC_3_18289
Diaporthe_ambigua_CBS_187_87
Diaporthe_ovalispora_ZJUD93
Diaporthe_sojae_FAU635_
Diaporthe_kochmanii_BRIP_54033
Diaporthe_sojae_MAFF_410444_
Diaporthe_infecunda_CBS_133812
Diaporthe_serafiniae_BRIP_55665a
Diaporthe_pyracanthae_CAA483
Diaporthe_sackstonii_BRIP_54669b
Diaporthe_serafiniae_BRIP_54136_
Diaporthe_mayteni_CBS_133185
Diaporthe_acaciarum_CBS_138862
Diaporthe_middletonii_BRIP_54884e
Diaporthe_middletonii_BRIP_57329_
Diaporthe_beilharziae_BRIP_54792T
Diaporthe_ueckerae_LGMF947_
Diaporthe_ueckerae_FAU658
Diaporthe_ueckerae_FAU656
Diaporthe_miriciae_BRIP_55662c_
Diaporthe_miriciae_BRIP_54736j
Diaporthe_tectonendophytica_MFLUCC_13_0471
Diaporthe_unshiuensis_ZJUD52
Diaporthe_unshiuensis_ZJUD50_
Diaporthe_longicicolla_FAU599
Diaporthe_melonis_CBS_507_78
Diaporthe_melonis_FAU640_
Diaporthe_passifloricola_CBS_141329
YI_005
OI_006
OO_005
YO_012
YI_002
OI_023
Diaporthe_masirevicii_BRIP_57892a_
Diaporthe_masirevicii_BRIP_54256_
Diaporthe_kongii_BRIP_54031
Diaporthe_endophytica_CBS_133811T
Diaporthe_paranensis_CBS_133184
Diaporthe_brasiliensis_CBS_133183
Diaporthe_caatingaensis_URM7485
Diaporthe_subclavata_ZJUD95_
Diaporthe_momicola_MFLUCC_16_0113_
Diaporthe_charlesworthii_BRIP54884m_
Diaporthe_oraccinii_LC3166_
Diaporthe_citrichinensis_ZJUD34_
Diaporthe_citrichinensis_ZJUD85_
Diaporthe_eres_DLR_12a_
Diaporthe_cotoneastri_CBS_439_82_
Diaporthe_asheicola_CBS_136967_
Diaporthe_lonicerae_MFLUCC_17_0963_
Diaporthe_eres_CBS_138598_
Diaporthe_eres_AR5193_
Diaporthe_phragmitis_CBS_138897_
Diaporthe_ellipicola_CGMCC3_17085_
Diaporthe_ellipicola_CGMCC3_17084_
Diaporthe_bicincta_CBS_121004_
Diaporthe_vaccinii_CBS_160_32_
Diaporthe_asheicola_CBS_136968
Diaporthe_vaccinii_CBS_122116_
Diaporthe_alleghaniensis_CBS_495_72
Diaporthe_celastrina_CBS_139_27_
Diaporthe_maritima_DAOMC_250563
Diaporthe_biguttusis_CGMCC_3_17082
Diaporthe_biguttusis_CGMCC_3_17081_
Diaporthe_apiculatum_LC3187_
Diaporthe_apiculatum_LC3418_
Diaporthe_longicicola_CGMCC_3_17090
Diaporthe_longicicola_CGMCC_3_17089
Diaporthe_penetriteum_LC3394_
Diaporthe_penetriteum_CGMCC_3_17532_
Diaporthe_penetriteum_LC3353_
Diaporthe_subclavata_ZJUD83_CGMCC_3_17253
Diaporthe_collariana_MFLUCC_17_2636
Diaporthe_vawdreyi_BRIP_57887a
Diaporthe_mahothocarpus_CGMCC3_15181_
Diaporthe_mahothocarpus_CGMCC3_15182
Diaporthe_fukushii_MAFF_625029
Diaporthe_neilliae_CBS_144_27_
Diaporthe_alnea_CBS_146_46_
Diaporthe_alnea_CBS_159_47_
Diaporthe_pulla_CBS_338_89_
Diaporthe_thunbergii_MFLUCC_10_576b_
Diaporthe_thunbergii_MFLUCC10_576a
Diaporthe_virgiliae_CMW_40748_
Diaporthe_rostrata_CFCC_50063_
Diaporthe_rostrata_CFCC_50062
Diaporthe_juglandicola_CFCC_51134
Diaporthe_sennicola_CFCC_51634_
Diaporthe_sennicola_CFCC_51635_
Diaporthe_betulicola_CFCC_51128
Diaporthe_betulicola_CFCC_51129
Diaporthe_passiflorae_CBS_132527
Diaporthe_citri_AR4469_
Diaporthe_citri_CBS_135422_
Diaporthe_oncostoma_CBS589_78
Diaporthe_perjuncta_CBS_109745
Diaporthe_ampelina_CBS_114016_
Diaporthe_ampelina_CBS_267_80_
Diaporthe_hickoriae_CBS_145_26_
Diaporthe_multigutullata_ZJUD_98
Diaporthe_undulata_CGMCC_3_18293
Diaporthe_nobilis_CBS_587_79_
Diaporthe_helicis_AR5211_
Phomopsis_fukushii_MAFF625029
Diaporthe_betulae_CFCC_50470
Diaporthe_betulae_CFCC_50469
Diaporthe_biconospora_ZJUD62
Diaporthe_biconospora_ZJUD61
Diaporthe_eucalyptorum_CBS_132525
Diaporthe_pterocarpicola_MFLUCC_10_0580a
Diaporthe_podocarpimacrophylli_CGMCC_3_18281
OI_008
OI_016
Diaporthe_hongkongensis_CBS_115448
OI_021
SI_007
Diaporthe_arengae_CBS_114979
Diaporthe_perseae_CBS_151_73
Diaporthe_sennae_CFCC_51636
Diaporthe_sennae_CFCC_51637_
Diaporthe_fraxini_angustifoliae_BRIP_54781T
Diaporthe_musigena_CBS_125919
Diaporthe_pascoei_BRIP_54847
Diaporthe_litchicola_BRIP_54900
Diaporthe_taoicola_MFLUCC_16_0117
Diaporthe_eugeniae_CBS444_82
Diaporthe_pseudomangiferae_CBS_101339
Diaporthe_pseudomangiferae_CBS_388_89_
Diaporthe_xishuangbanica_CGMCC_3_18282
Diaporthe_tectonigena_MFLUCC_12_0767
SO_003
OO_014
Diaporthe_pescicola_MFLUCC_16_0105
Diaporthe_pseudophoenicicola_CBS_462_69
Diaporthe_arecae_CBS161_64
Diaporthe_pseudophoenicicola_CBS_176_77_
Diaporthe_aseana_MFLUCC_12_0299a
RI_004
OO_016
Diaporthe_lithocarpus_CGMCC_3_15178
Diaporthella_corylina_outgroup_CBS_121124_
3.0

Supplementary Figure S3. Phylogenetic tree of 66 isolates based on the nucleotide sequences of rDNA-ITS The isolates and their phylogenetic position based on the ITS region according to the RAxML analyses. Bootstrap support values for ML equal to or greater than 90%.

Supplementary Table S1. Maximum nucleotide identity matches for 66 fungal isolated based on ITS sequences using BLAST and phylogenetic tree analysis.

No.	Isolates ID	GenBank accession number	Database species	Identity (%)	Accession number	Phylum, Class, Family
1	YI-002	OM189555	*Diaporthe sp.*	96.54%	MT043777.1	Ascomycota, Sordariomycetes, Nectriaceae,
2	YI-005	OM190460	*Diaporthe sp.*	99.59%	KP199195.1	Ascomycota, Sordariomycetes, Diaporthaceae
3	YI-008	OM190401	*Pleosporales sp.*	99.48%	HQ832808.1	Ascomycota, Sordariomycetes, Diaporthaceae
4	YI-012	OM149730	*Neopestalotiopsis sp.*	99.20%	MT576586.1	Ascomycota, Sordariomycetes, Amphisphaeriaceae
5	OI-004	OM190461	*Fusarium oxysporum*	99.81%	MT560381.1	Ascomycota, Sordariomycetes, Diaporthaceae
6	OI-006	OM127751	*Diaporthe sp.*	99.29%	KF498865.1	Ascomycota, Sordariomycetes, Amphisphaeriaceae
7	OI-008	OM127832	*Diaporthe sp.*	98.92%	MG832475.1	Ascomycota, Sordariomycetes, Diaporthaceae
8	OI-010	OM127833	*Pseudopestalotiopsis sp.*	99.82%	MT322075.1	Ascomycota, Sordariomycetes, Diaporthaceae
9	OI-016	OM127847	*Diaporthe sp.*	98.75%	MG832475.1	Ascomycota, Sordariomycetes, Chaetomiaceae
10	OI-019	OM127834	*Pseudopestalotiopsis sp.*	83.25%	MT322091.1	Ascomycota, Sordariomycetes, Diaporthaceae
11	OI-021	OM190463	*Diaporthe sp.*	97.84%	KY790594.1	Ascomycota, Dothideomycetes Didymosphaeriaceae
12	OI-023	OM127848	*Diaporthe sp.*	92.73%	MK111104.1	Ascomycota, Sordariomycetes, Chaetomiaceae
13	SI-002	OM189550	*Neopestalotiosis sp.*	100%	MN486554.1	Ascomycota, Eurotiomycetes, Trichocomaceae.

14	SI-007	OM108436	*Diaporthe sp.*	98.53%	KY790594.1	Ascomycota, Sordariomycetes, Chaetomiaceae
15	SI-012	OM189548	*Clonostachys sp.*	100%	MN486561.1	Ascomycota, Sordariomycetes, Xylariaceae
16	SI-013	OM189547	*Neopestalotiopsis sp.*	99.63%	MT151848.1	Ascomycota, Sordariomycetes, Diaporthaceae
17	SI-015	OM117569	*Neopestalotiopsis sp.*	100%	LC412067.1	Ascomycota, Sordariomycetes, Bionectriaceae
18	SI-016	OM135584	*Neopestalotiopsis sp.*	99.41%	MK278906.1	Ascomycota, Sordariomycetes, Sporocadaceae
19	SI-017	OM117591	*Neopestalotiopsis sp.*	100%	MT322103.1	Ascomycota, Sordariomycetes, Sporocadaceae
20	RI-003	OM124054	*Chaetomium sp.*	98.65%	MN341327.1	Ascomycota, Sordariomycetes, Sporocadaceae
21	RI-004	OM135374	*Diaporthe aseana*	84.58%	KF159984.1	Ascomycota, Sordariomycetes, Sporocadaceae
22	RI-005	OM124055	*Roussoella sp.*	99.40%	MT112308.1	Ascomycota, Sordariomycetes, Diaporthaceae
23	RI-007	OM124056	*Chaetomium sp.*	97.61%	MH855939.1	Ascomycota, Sordariomycetes, Diaporthaceae
24	RI-008	OM124069	*Eurotium sp.*	99.81%	KJ863518.1	Ascomycota, Dothideomycetes, Pleosporomycetidae
25	RI-012	OM169009	*Ovatospora sp.*	99.64%	MN886594.1	Ascomycota, Sordariomycetes, Sporocadaceae
26	YO-002	OM125263	*Phaeosphaeriopsis sp.*	99.42%	KU529845.1	Ascomycota, Sordariomycetes, Diaporthaceae
27	YO-004	OM190448	*Diaporthe sp.*	99.09%	MN816409.1	Ascomycota, Sordariomycetes, Nectriaceae,
28	YO-007	OM190449	*Neopestalotiopsis sp.*	99.81%	MT019206.1	Ascomycota, Dothideomycetes, Pleosporomycetidae

29	YO-008	OM125357	*Phaeosphaeriopsis sp.*	98.57%	KM434268.1	Ascomycota, Dothideomycetes Pleosporaceae
30	YO-010	OM190441	*Neopestalotiopsis sp.*	99.81%	MT151848.1	Ascomycota, Sordariomycetes, Trichosphaeriaceae
31	YO-012	OM190454	*Diaporthe sp.*	99.64%	MT043777.1	Ascomycota, Sordariomycetes, Diaporthaceae
32	YO-020	OM200353	*Cladosporium sp.*	87.21%	MT645944.1	Ascomycota, Sordariomycetes, Diaporthaceae
33	YO-024	OM190458	*Pseudopestalotiopsis sp.*	100 %	MK909901.1	Ascomycota, Dothideomycetes Pleosporaceae
34	YO-025	OM190457	*Nigrospora sp.*	100%	MW186168.1	Ascomycota, Sordariomycetes, Glomerellaceae
35	YO-026	OM190453	*Neopestalotiopsis sp.*	99.81%	MT151848.1	Ascomycota, Sordariomycetes, Amphisphaeriaceae
36	YO-027	OM200315	*Nigrospora sp.*	99.81%	MW186168.1	Ascomycota, Sordariomycetes, Nectriaceae
37	OO-005	OM190402	*Diaporthe sp.*	99.65%	FJ79938.1	Ascomycota, Sordariomycetes, Apiosporaceae
38	OO-008	OM189553	*Fusarium sp.*	100%	MT928791.1	Ascomycota, Sordariomycetes, Nectriaceae,
39	OO-009	OM190403	*Pleosporales sp.*	99.65%	HQ832808.1	Ascomycota, Dothideomycetes Didymosphaeriaceae
40	OO-010	OM108317	*Alternaria sp.*	100%	MH884141.1	Ascomycota, Sordariomycetes, Nectriaceae,
41	OO-012	OM131564	*Nigrospora sp.*	99.60%	MH979024.1	Ascomycota, Sordariomycetes, Xylariaceae
42	OO-014	OM131565	*Diaporthe sp.*	98.30%	KX940974.1	Ascomycota, Dothideomycetes, Pleosporomycetidae
43	OO-016	OM189552	*Diaporthe sp.*	99.27%	NR_154920.1	Ascomycota, Sordariomycetes, Bionectriaceae

44	OO-018	OM131721	*Alternaria sp.*	100%	MH884078.1	Ascomycota, Sordariomycetes, Diaporthaceae
45	OO-019	OM189551	*Colletotrichum sp.*	99.82%	KT218694.1	Ascomycota, Dothideomycetes, Pleosporomycetidae
46	SO-001	OM189543	*Clonostachys sp.*	100%	MN486561.1	Ascomycota, Sordariomycetes, Amphisphaeriaceae
47	SO-003	OM117688	*Diaporthe sp.*	99.45%	MN651490.1	Ascomycota, Dothideomycetes, Botryosphaeriaceae
48	SO-006	OM149384	*Pleosporales sp.*	99.82%	JQ809679.1	Ascomycota, Sordariomycetes, Trichosphaeriaceae
49	SO-007	OM149386	*Pseudopestalotiopsis sp.*	97.61%	MT322085.1	Ascomycota, Dothideomycetes, Pleosporomycetidae
50	SO-010	OM149387	*Guignardia sp.*	100%	EU677801.1	Ascomycota, Sordariomycetes, Nectriaceae,
51	SO-011	OM149390	*Nigrospora sp.*	98.59%	MH979024.1	Ascomycota, Dothideomycetes, Pleosporomycetidae
52	SO-013	OM149392	*Pleosporales sp.*	97.56%	HQ832808.1	Ascomycota, Sordariomycetes, Sporocadaceae
53	SO-014	OM190412	*Fusarium sp.*	99.46%	KF918554.1	Ascomycota, Dothideomycetes, Davidiellaceae
54	SO-015	OM149713	*Pleosporales sp.*	98.95%	HQ832808.1	Ascomycota, Dothideomycetes, Pleosporomycetidae
55	SO-019	OM190406	*Neopestalotiopsis sp.*	99.81%	MT151848.1	Ascomycota, Sordariomycetes, Valsaceae
56	SO-022	OM149718	*Cladosporium sp.*	99.02%	MG209675.1	Ascomycota, Dothideomycetes, Phaeosphaeriaceae
57	SO-024	OM190404	*Pleosporales sp.*	89.93%	HQ832808.1	Ascomycota, Sordariomycetes, Diaporthaceae
58	SO-025	OM189554	*Phomopsis sp.*	99.74%	MF800892.1	Ascomycota, Sordariomycetes, Sporocadaceae

59	RO-001	OM190466	*Pseudopestalotiopsis sp.*	99.63%	MK909901.1	Ascomycota, Dothideomycetes, Phaeosphaeriaceae
60	RO-002	OM108320	*Fusarium sp.*	100%	MT928794.1	Ascomycota, Sordariomycetes, Sporocadaceae
61	RO-004	OM108434	*Arthrinium sp.*	91.33%	NR166043.1	Ascomycota, Sordariomycetes, Diaporthaceae
62	RO-005	OM149376	*Fusarium sp.*	92.68%	MT560381.1	Ascomycota, Dothideomycetes, Davidiellaceae
63	RO-007	OM112302	*Roussoella sp.*	96.93%	MT112308.1	Ascomycota, Sordariomycetes, Amphisphaeriaceae
64	RO-009	OM190483	*Fusarium sp.*	99.81	KP050556.1	Ascomycota, Sordariomycetes, Trichosphaeriaceae
65	RO-010	OM135271	*Hypoxylon sp.*	93.42%	KC968937.1	Ascomycota, Sordariomycetes, Sporocadaceae
66	RO-013	OM123443	*Pleosporales sp.*	99.45%	JQ809679.1	Ascomycota, Sordariomycetes, Trichosphaeriaceae
